# MicroRNA-150 Expression Induces Myeloid Differentiation of Human Acute Leukemia Cells and Normal Hematopoietic Progenitors

**DOI:** 10.1371/journal.pone.0075815

**Published:** 2013-09-24

**Authors:** Valerie A. Morris, Ailin Zhang, Taimei Yang, Derek L. Stirewalt, Ranjani Ramamurthy, Soheil Meshinchi, Vivian G. Oehler

**Affiliations:** 1 Clinical Research Division, Fred Hutchinson Cancer Research Center, Seattle, Washington, United States of America; 2 Division of Hematology, Department of Medicine, University of Washington, Seattle, Washington, United States of America; 3 Department of Medicine, University of Washington, Seattle, Washington, United States of America; B.C. Cancer Agency, Canada

## Abstract

In acute myeloid leukemia (AML) and blast crisis (BC) chronic myeloid leukemia (CML) normal differentiation is impaired. Differentiation of immature stem/progenitor cells is critical for normal blood cell function. MicroRNAs (miRNAs or miRs) are small non-coding RNAs that interfere with gene expression by degrading messenger RNAs (mRNAs) or blocking protein translation. Aberrant miRNA expression is a feature of leukemia and miRNAs also play a significant role in normal hematopoiesis and differentiation. We have identified miRNAs differentially expressed in AML and BC CML and identified a new role for miR-150 in myeloid differentiation. Expression of miR-150 is low or absent in BC CML and AML patient samples and cell lines. We have found that expression of miR-150 in AML cell lines, CD34+ progenitor cells from healthy individuals, and primary BC CML and AML patient samples at levels similar to miR-150 expression in normal bone marrow promotes myeloid differentiation of these cells. MYB is a direct target of miR-150, and we have identified that the observed phenotype is partially mediated by MYB. In AML cell lines, differentiation of miR-150 expressing cells occurs independently of retinoic acid receptor α (RARA) signaling. High-throughput gene expression profiling (GEP) studies of the AML cell lines HL60, PL21, and THP-1 suggest that activation of CEPBA, CEBPE, and cytokines associated with myeloid differentiation in miR-150 expressing cells as compared to control cells contributes to myeloid differentiation. These data suggest that miR-150 promotes myeloid differentiation, a previously uncharacterized role for this miRNA, and that absent or low miR-150 expression contributes to blocked myeloid differentiation in acute leukemia cells.

## Introduction

Acute leukemia is characterized by increased self-renewal of leukemia stem/progenitor cells, decreased cell death, and a block in differentiation that retains cells at an immature blast stage with limited to no capacity to produce mature cells [1,2]. MiRNAs are small single-stranded non-coding RNAs 19 to 24 nucleotides in length that regulate expression of tens to hundreds of genes via mRNA degradation or translational repression, based on the sequence complementarity to the miRNA seed region (positions 2-8) [3,4]. In myeloid differentiation, the coordinated expression of transcription factors such as PU.1, CEBPA, CEBPE, GFI1, EGR2, NAB2, and IRF8 regulate differentiation from early myeloid progenitors to terminally differentiated granulocytes or monocyte/macrophages [5-7]. Recent evidence suggests that microRNAs (miRNAs or miRs) are important regulators of normal hematopoietic differentiation [8-13]. Transcription factors such as PU.1 regulate a number of miRNAs, and correspondingly, miRNAs also regulate transcription factor expression in normal hematopoiesis [11,12,14,15]. For example, miR-223 and the transcription factors CEPBA, PU.1, NFI-A and E2F2 contribute to a regulatory circuit that controls normal granulopoiesis [16,17]. Studies of primary leukemia samples have identified miRNAs that are differentially expressed in acute myeloid leukemia (AML) or blast crisis (BC) chronic myeloid leukemia (CML) as compared to normal hematopoietic progenitor cells [18,19]. Recent functional investigations have begun to identify how aberrant miRNA expression contributes to impaired differentiation in acute leukemia. These studies have described contributions by miR-328 (BC CML), miRs-29a and 142-3p (AML), miR-9 (EVI1-induced AML), and miR-193a (AML associated with t(8;21)) [20-23]. In several cases a previously uncharacterized role for these miRNAs in normal myelopoiesis has also been identified that is altered in AML [21,22].

MiRNA expression profiling studies of sorted normal hematopoietic cell populations have demonstrated that miR-150 is expressed in normal stem/progenitor cells [10]. Two studies have reported that miR-150 expression is decreased in advanced phase CML patients [24,25], but the impact of low expression in BC CML has been unknown. In AML, two high-throughput miRNA expression studies of 110 patient samples have identified that miR-150 expression is decreased in AML patient samples with various cytogenetic abnormalities [18,26]. Expression in normal karyotype (NK) AML is heterogeneous, but many NK AML samples also demonstrate low miR-150 expression [26,27]. These studies, in addition to our own studies presented here of 182 pediatric and 22 adult AML patients, and 10 BC CML patients suggest downregulation of miR-150 is a pervasive finding in acute myeloid leukemias [18,26].

MiR-150 influences cell fate decisions in megakaryocytic-erythroid progenitors and lymphoid cells, and contributes to natural killer cell development [9,28-30]. No role in normal myeloid differentiation has been described for miR-150. It has also been identified as a tumor suppressor in lymphoma cells [31]. In lymphoid and megakaryocytic lineage commitment the effects of miR-150 are primarily mediated by regulation of the transcription factor MYB [9,28]. Recently, a role for miR-150 was identified in MLL-rearranged AML. The repression of miR-150 maturation by MLL-fusion genes accelerated leukemogenesis in an MLL-AF9 murine model and miR-150 expression in this model inhibited leukemia cell growth [27]. Given that in hematopoietic progenitor cells, MYB expression is high and its expression is down-regulated during terminal differentiation [32,33], we hypothesized that low or absent miR-150 expression may contribute to impaired myeloid differentiation. We now report that miR-150 expression promotes myeloid differentiation of various AML cell lines, primary human BC CML and AML patient cells, and CD34+ progenitor cells from healthy individuals, in part through regulation of MYB. This is a previously uncharacterized role for miR-150 in myeloid differentiation and suggests that low miR-150 expression contributes to the leukemic phenotype in various AML subtypes and BC CML.

## Results

### MiR-150 expression is decreased in BC CML and AML patient cells and cell lines compared to unsorted normal bone marrow

**Figure 1:**
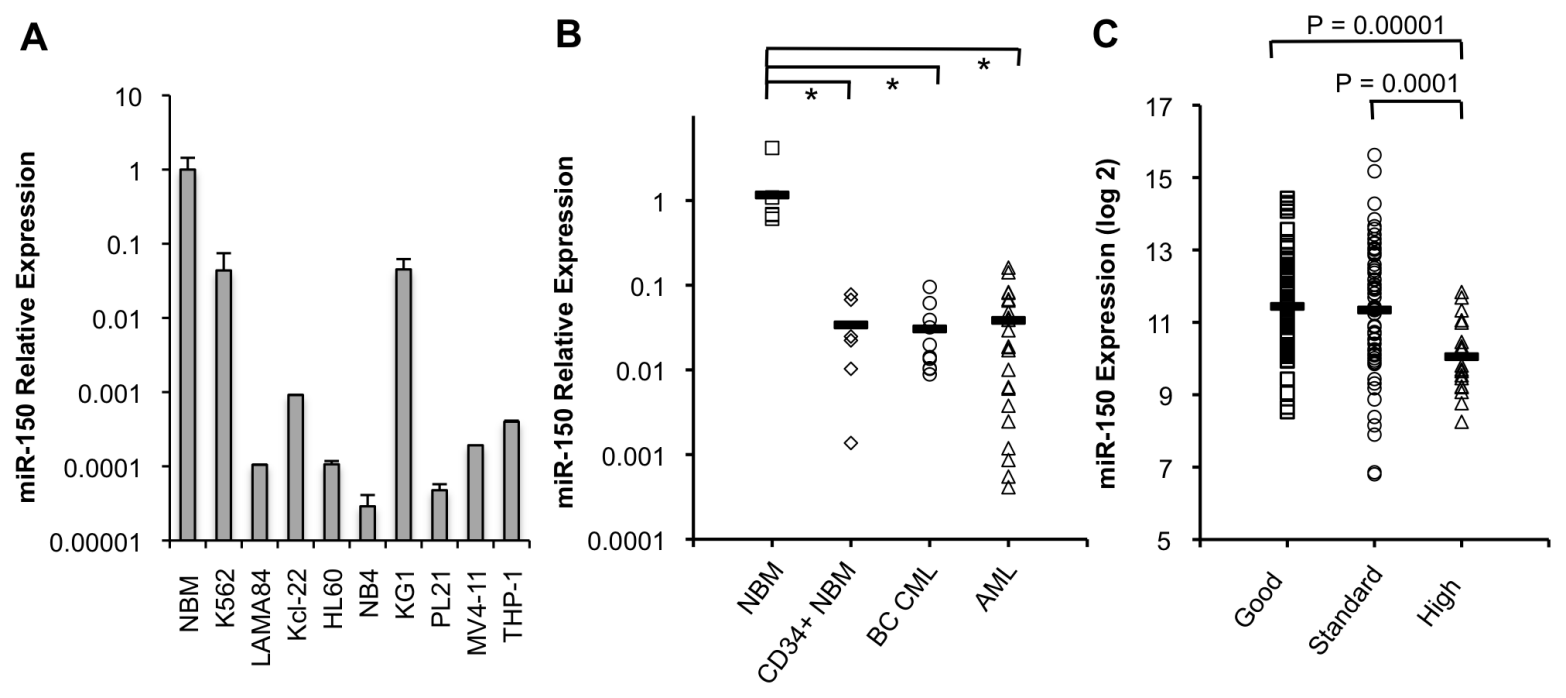
Mature miR-150 expression is low in myeloid leukemia cells. Mature miR-150 expression is decreased in CML and AML cell lines and in primary patient CD34+ progenitor cells from healthy individuals, BC CML and AML samples relative to average miR-150 expression in normal bone marrow (NBM). (A) MiR-150 expression is low or absent in BC CML (K562, LAMA84, and Kcl-22) and AML (HL60, NB4, KG1, PL21, MV4-11, and THP-1) cell lines. Expression is shown as a fold-change relative to miR-150 expression in NBM on a log_10_ scale as determined by QPCR (performed in duplicate). (B) By QPCR, miR-150 expression was decreased in CD34+ sorted (n=6) vs. unsorted (n=5) NBM. In primary BC CML (n=10) and adult AML (n=22) patient samples, miR-150 expression was significantly decreased compared to unsorted NBM, but not sorted CD34+ NBM. Individual patient samples are shown as fold-change relative to miR-150 expression in NBM on a log_10_ scale, with means indicated by bars (*P≤0.05, **P≤0.001 for comparisons, Student’s t-test). (C) MiR-150 expression was obtained by RNA sequencing for 182 pediatric AML cases and was stratified by risk group based on cytogenetic and molecular abnormalities. Expression is on a log_2_ scale, with averages indicated by bars (Student’s t-test used for comparisons).

We examined mature miR-150 expression by quantitative PCR (QPCR) in cell lines, normal bone marrow (NBM, N=5), CD34+ sorted normal bone marrow (N=6), myeloid BC CML (N=10), and adult AML (N=22) patient samples. Patient characteristics are shown in Table **S1**. By QPCR, miR-150 expression was low or absent in 9 CML and AML cell lines compared to NBM (Figure **1A**). MiR-150 expression was significantly lower in CD34+ sorted progenitor cells from healthy individuals and in adult BC CML and AML leukemia samples as compared to average miR-150 expression in unsorted NBM (Figure **1B**). The lowest expression was observed in a subset of AML patient samples where expression was 100 to 1000-fold lower than NBM.

In order to assess how miR-150 expression varies with myeloid differentiation in normal cells we examined data from a study that measured the expression of various miRNAs, including miR-150, in multiple murine hematopoietic compartments using QPCR [10]. Our analysis of these data for miR-150 is shown in Figure **S1A** and demonstrates that miR-150 is moderately expressed in stem cell populations, decreases in expression as cells mature into common myeloid and granulocyte/macrophage progenitors, but increases again as cells differentiate into granulocytes, monocytes, and macrophages. In keeping with these observations in human mobilized CD34+ peripheral blood stem/progenitor cells (PBSCs) from two healthy individuals cultured *in vitro* we observed that miR-150 expression increases as these cells differentiate in response to G-CSF and GM-CSF (Figure **S1B**).

We then extended our examination of miR-150 expression in 182 pediatric AML cases using RNA-Seq (Illumina HiSeq 2000, Figure **1C**). Pediatric AML patient characteristics are shown in Table **S2A**. No relationship between miR-150 expression and age, gender, or FAB classification was observed, and no significant correlation was found between miR-150 expression and blast percentages in pediatric or adult cases. For pediatric patients, 144 cases had evaluable karyotype data; and 180, 171, 176, and 175 patients were evaluable for FLT3 internal tandem duplications (ITD) and NPM1, CEBPA, and WT1 mutations, respectively. Lower miR-150 expression was associated with FLT3 ITD positive and NPM1 mutation positive status. Using accepted definitions of good, standard, and high risk AML [34], lower miR-150 expression was associated with high risk AML (Figure **1C** and Table **S2B**). In the adult AML patients a similar association between miR-150 expression and poor risk cytogenetic status was observed; however, these observations did not reach statistical significance in this smaller data set.

### MiR-150 expression in AML cell lines promotes myeloid differentiation

Based on our observations of decreased miR-150 expression in leukemia cells as compared to NBM, increasing miR-150 expression with myeloid differentiation of CD34+ progenitors, and the role of miR-150 in some cell fate decisions, we hypothesized that expression of miR-150 may alter myeloid differentiation. For these studies we examined AML cell lines (HL60, NB4, PL21, and THP-1) that can be induced to differentiate along the granulocytic or monocytic lineage after exposure to drugs such as all-trans retinoic acid (ATRA) or 12-O-tetra-decanoylphorbol-13-acetate (TPA), respectively. As shown in Figure **1A**, NB4, HL60, PL21 and THP-1 cells express very low levels of endogenous miR-150. In contrast to normal CD34+ progenitors exposed to cytokines (Figure **S1B**), we did not observe that miR-150 expression increased significantly in cell lines after exposure to ATRA or TPA (data not shown).

**Figure 2:**
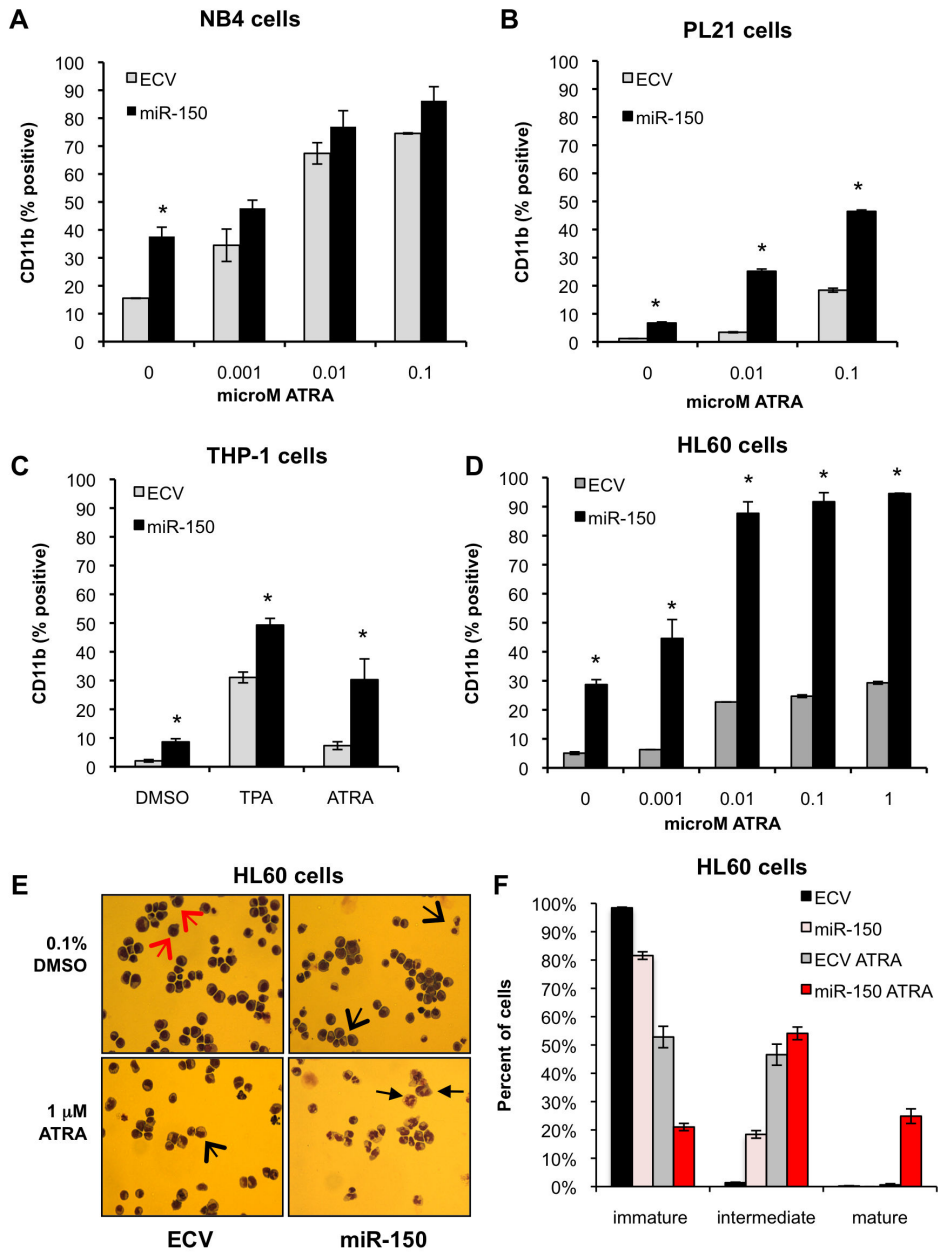
MiR-150 expression promotes myeloid differentiation in AML cell lines. AML cell lines NB4, PL21, HL60, and THP-1 were transduced with miR-150 or empty control lentiviral (ECV) supernatants, flow sorted for GFP, and assayed 7-21 days post transduction. (A) NB4 cells, an acute promyelocytic leukemia (APL) cell line with the t(15;17) PML-RARA translocation, were treated with the indicated concentrations of ATRA or vehicle control (0.1% DMSO) for 72 hours and assayed by flow cytometry for CD11b expression, displayed as percentage of CD11b positive cells. (B) PL21 cells, an APL cell line with FLT3 ITD, were assayed after 96 hours of ATRA treatment. (C) THP-1 cells, a MLL-AF9 rearranged monocytic AML cell line, were treated with vehicle control (0.1% DMSO) or TPA (12.5 ng/mL) for 24 hours, or DMSO or ATRA (1 µM) for 48 hours and then assayed. (D) HL60 cells, an AML cell line, were assayed after 96 hours of ATRA treatment. For all cell lines assayed, miR-150 expression induced CD11b expression in the absence or presence of TPA or ATRA. For all experiments the means of three independent experiments each performed in triplicate are shown; error bars represent standard deviations (*P≤0.*05*, ** *P≤0.005* for comparisons, Student’s t-test). (E) HL60 cells expressing miR-150 displayed increased morphological evidence of differentiation by Wright-Giemsa staining, most notable after exposure to 1 µM ATRA for 96 hours. These features included decreased nuclear to cytoplasmic ratio, lobulated nuclei, and granules. Immature cells are indicated by the large red arrows, intermediate differentiated cells are indicated by the large black arrows and mature myeloid cells by the small black arrows. (F) Quantitation of cells in different stages of myeloid differentiation was determined by Wright-Giemsa stain as described in Methods. Percentage of cells in each stage of differentiation displayed as an average of 10 fields (400 cells counted), error bars indicate standard deviations.

Pre-miR-150 was cloned into a lentiviral vector under the control of a MSCV promoter with GFP under the control of a PGK promoter [35]. HL60, NB4, PL21, and THP-1 cell lines were transduced with empty vector control or pre-miR-150 and flow sorted for GFP-positive cells. Mature miR-150 was expressed at levels similar to its expression in NBM (Figure **S2A**). Sorted cells were treated with vehicle control (0.1% DMSO) or ATRA at varying concentrations for 48-96 hours and assayed by flow cytometry for CD11b expression, a marker of myeloid differentiation, and by Wright-Giemsa staining to assess cell morphology. In all cell lines examined, cells expressing pre-miR-150 demonstrated increased expression of CD11b as compared to control cells (Figure **2**). Notably this increase in CD11b was observed not only after exposure to ATRA, but also in the absence of differentiating agents. We also observed morphological evidence of increased differentiation by Wright-Giemsa staining (HL60 shown, Figure **2E, F**). Similar differences in CD11b expression were also observed in miR-150 expressing THP-1 cells after exposure to TPA (Figure **2C**). Cell proliferation was also decreased in miR-150 expressing cells (Figure **S3**)**.**

Additionally, as another means to confirm myeloid differentiation, we utilized high-throughput gene expression profiling (GEP) (Illumina Human HT-12 v. 4 microarrays) to assess enrichment of genes associated with myeloid differentiation and to elucidate potential pathways by which miR-150 promotes myeloid differentiation. For these studies we compared HL60, PL21, and THP-1 cells transduced with miR-150 or control vector. The most statistically significant differentially expressed genes are shown in Table **S3**. Ingenuity Pathways Analysis (IPA, Ingenuity Systems®, www.ingenuity.com) consistently identified functions and pathways associated with myeloid cells and myeloid differentiation. The top functions enriched in miR-150 versus control cells included cell movement of myeloid cells or neutrophils (*P*=8.16E-12 and *P*=1.10E-12), activation of myeloid cells (*P*=1.34E-8), respiratory burst (*P*=1.31E-14), production of superoxide (*P*=1.20E-8), degranulation of cells (*P*=7.07E-9) and adhesion of granulocytes or phagocytes (*P*=3.93E-8 and 1.69E-8). We also compared these data to a gene signature associated with myeloid differentiation of AML cells [36-38]. Consistent with myeloid differentiation we observed increased expression of CD14, EMR3, NCF1, NCF2, SLCA3, S100A8, and S100A9, which was confirmed by QPCR (Figure **S4**).

Recent reports suggest that in certain cases the partner star (*) miRNA strands, rather than being degraded, are also functional and target similar or different mRNAs based on their seed sequences [39,40]. Since pre-miR-150 transduction results in both mature miR-150 and miR-150* expression in the AML cell lines (Figure **S2A**), we examined whether mature miR-150, miR-150*, or both promote myeloid differentiation. The seed sequences of both forms were mutated individually or in combination in the original lentiviral vector construct (Figure **S5**). Notably, transduction of the mutated mature miR-150 or double mutant vectors did not induce differentiation, whereas the mutated miR-150* vector was still able to increase CD11b expression in HL60 and NB4 cells. These results indicate that miR-150 targets, but not miR-150* targets, are likely responsible for promoting myeloid differentiation in these cells. Together, these results demonstrate that mature miR-150 expression promotes myeloid differentiation in myeloid leukemia cell lines.

### MiR-150 expression in CD34+ progenitors from healthy individuals promotes myeloid differentiation

**Figure 3:**
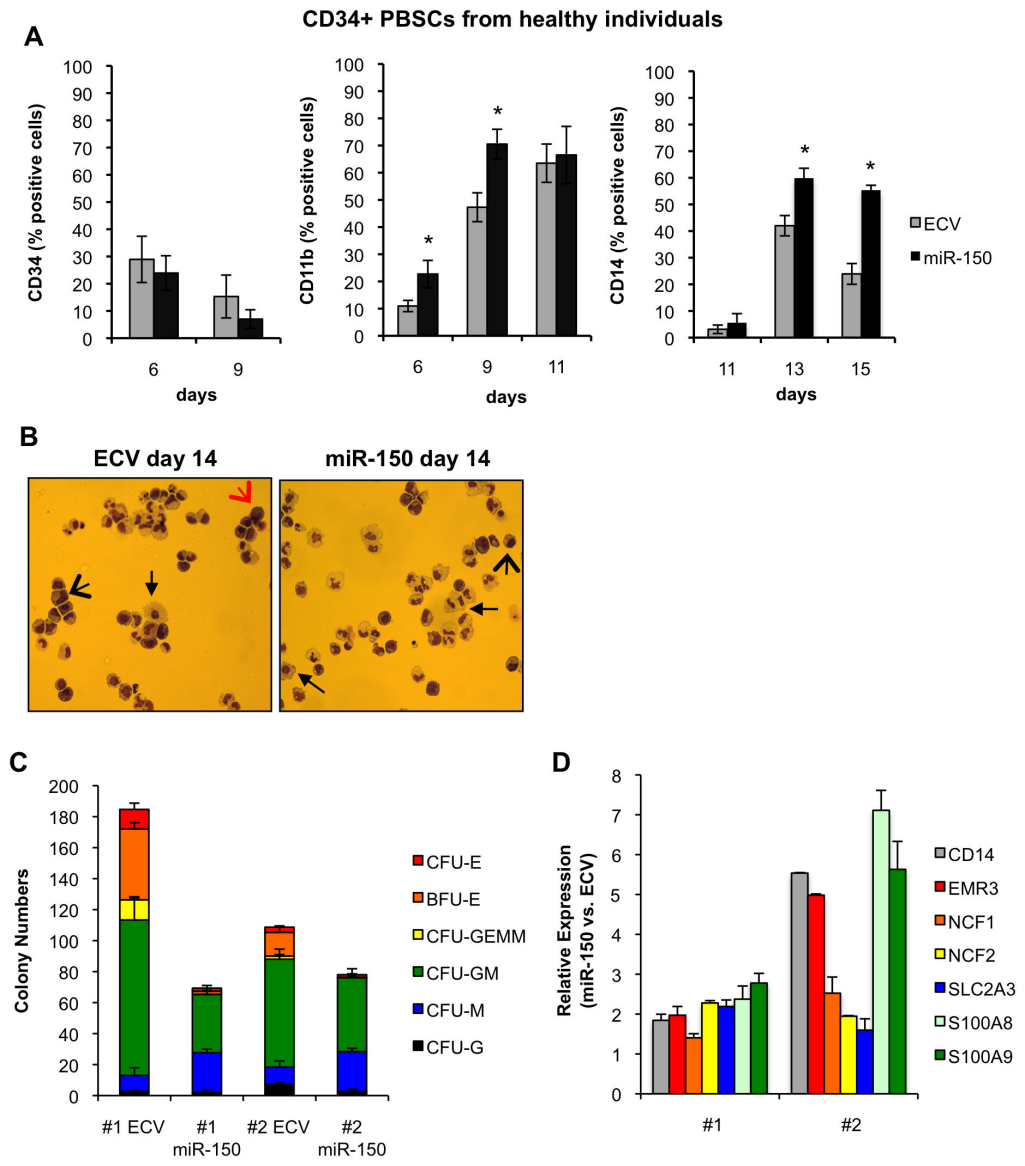
MiR-150 expression promotes myeloid differentiation of CD34+ PBMCs from healthy donors. Primary CD34+ PBMCs were transduced with pre-miR-150 or empty control lentiviral (ECV) supernatants, sorted for GFP expression, and then assayed by flow cytometry for CD11b, CD34, and CD14 expression or colony formation by colony-forming unit (CFU) assays. (A) Cells were assessed by flow cytometry at the indicated days after cell transduction cultured in the presence of rhSCF, rhIL3, rhIL6, rhGCSF, rhGMCSF (50 ng/ml each), and EPO (2 U/ml). CD11b and CD14 expression increased in both patient samples in the miR-150 vs. control transduced cells, while CD34 did not significantly change. The means from combined patients each run in duplicate experiments are shown; the error bars represent standard deviations (*P≤0.*05*, for comparisons, Student’s t-test). (B) At day 14 after transduction, miR-150 or ECV transduced CD34+ PBSC were stained with Wright-Giemsa after cytospin preparations and showed morphological evidence of myeloid differentiation, including immature cells (large red arrows), intermediate differentiated cells (large black arrows), and mature myeloid cells (small black arrows). (C) Cells were sorted and plated in triplicate 3 days post transduction in Methocult™ containing 20% lot-tested FBS, 10% BSA, and cytokine concentrations as above. Colonies were counted 12-16 days after plating. The mean colony numbers from triplicate plates are shown for each patient sample (#1 or #2); error bars indicate standard deviations. MiR-150 transduced cells had significantly lower colony numbers compared to control transduced cells, with decreased erythroid colonies (BFU-E and CFU-E), decreased CFU-G and CFU-GM, but increased monocyte/macrophage colonies (CFU-M) (*P≤0.05* for comparisons, Student’s t-test). (D) Expression of genes associated with myeloid differentiation was assessed in both CD34+ PBSC patient samples transduced with either miR-150 or ECV 9 days after transduction by QPCR. Relative fold-difference in expression for miR-150 vs. ECV cells is displayed for each patient sample; error bars represent standard deviations of technical duplicates.

We hypothesized that miR-150 may play a previously uncharacterized role in normal myeloid differentiation based upon our observations in primary patient cells and in AML cell lines. To assess if miR-150 induced differentiation of normal progenitor cells, mobilized CD34+ PBSCs from two healthy individuals were transduced with pre-miR-150 or control vector and sorted for GFP expression 3 days after transduction [35]. Mature miR-150 was expressed at levels approximating expression in NBM cells (Figure **S2B**). After transduction, cells were cultured in media with cytokines (Methods and Methods **S1**) or plated in colony forming unit (CFU) assays. In general normal CD34+ progenitors terminally differentiate in culture in response to cytokines such as G-CSF and GM-CSF over a 10-14 day period, with decreasing CD34 expression and increasing CD11b and CD14 expression [41]. To assess myeloid differentiation of cells in suspension culture, we examined CD34, CD11b, and CD14 expression by flow cytometry over time. Increased miR-150 expression increased CD11b and CD14 expression in both samples (Figure **3A**). Additionally, while the percent of CD11b positive cells reached maximum, mean fluorescence intensity for CD11b continued to increase further over time in miR-150 over-expressing cells versus control cells (data not shown). Depending on time in culture the maximum absolute increases in percent CD11b or CD14 expression ranged from ~15% to 30% in miR-150 over-expressing cells vs. ECV cells, reflecting a 1.5 to 2.4-fold increase in CD11b or CD14 expression in miR-150 cells vs. control cells. We also observed a trend towards decreased CD34 expression in miR-150 over-expressing cells vs. control cells. Notably, CD34 expression decreases rapidly during the transduction period itself, which may limit our ability to detect differences. Evidence of myeloid differentiation was observed morphologically for both control and miR-150 expressing cells (Figure **3B**). In Methocult assays, miR-150 expression decreased total CFU formation in both patient samples (Figure **3C**). These CFU data suggest that miR-150 expression inhibits colony-forming capacity either as a consequence of decreased cell proliferation or because miR-150 expressing cells are more differentiated and therefore less capable of forming myeloid colonies. Myeloid CFU-G and CFU-GM (*P*<0.01), erythroid BFU-E and CFU-E (*P*<0.01), and mixed CFU-GEMM (*P*<0.05) were significantly decreased in miR-150 expressing versus control cells. This observation is in agreement with previously published work suggesting that miR-150 modulates erythroid differentiation [9]. Notably, there was a significant increase in CFU-M colonies in miR-150 expressing (25.7±2.0) versus control cells (10.7±4.0, *P*<0.01). Lastly, we found increased expression of genes associated with granulocytic and monocytic differentiation and, in particular, as in the AML cell lines, large increases in S100A8 and S100A9, which are highly expressed in the cytosol of granulocytes and monocytes (Figure **3D**) [36-38]. Together, these data suggest that increased expression of miR-150 promotes myeloid differentiation of normal CD34+ progenitor cells and may favor an increase in monocytic differentiation.

### MiR-150 expression in primary AML and BC CML patient cells promotes myeloid differentiation

**Figure 4:**
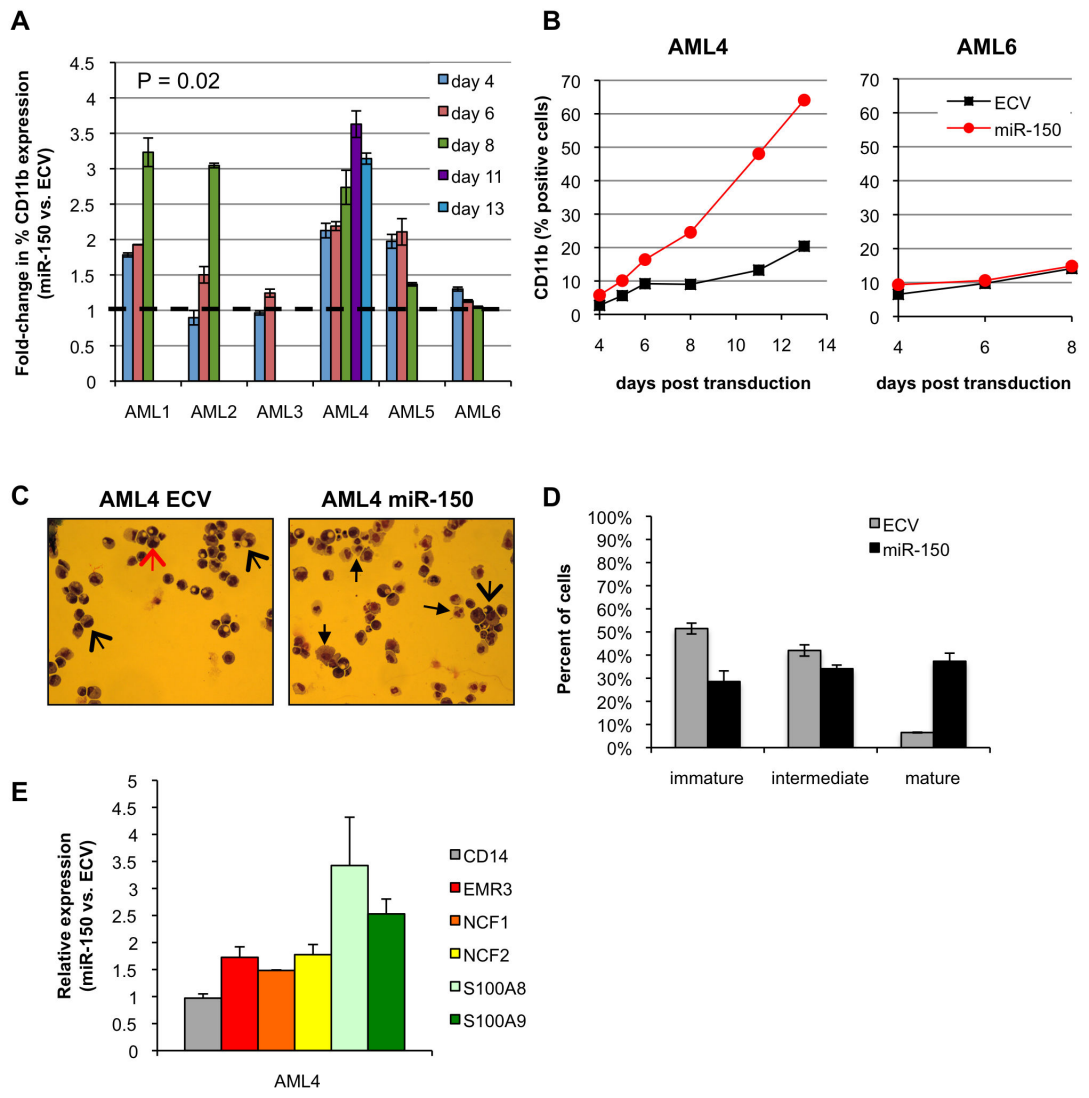
MiR-150 expression induces myeloid differentiation in primary AML cells. Six primary AML patient samples were transduced with pre-miR-150 or empty control lentiviral (ECV) supernatants, sorted for GFP expression, and then assayed by flow cytometry (performed in duplicate) for CD34 and CD11b expression or colony formation by colony-forming unit (CFU) assays. Cells were assessed in presence of rhSCF, rhIL3, rhIL6, rhGCSF, and rhGMCSF (50 ng/ml each) (A) The fold-change increase in CD11b expression in miR-150 expressing cells vs. control cells is displayed individually for 6 AML patient samples. The dotted line indicates the expectation if no change is observed (one sample t-test, P=0.02). (B) Two representative AML patient sample examples of CD11b expression (% positive cells) over time. (C) MiR-150 vs. ECV AML4 patient cells were stained with Wright-Giemsa 12 days after transduction. Arrows indicate immature cells (large red arrows), intermediate differentiated cells (large black arrows), and mature myeloid cells (small black arrows). (D) The following 3-group strategy was used to quantitate cells in various stages of myeloid differentiation: blasts (immature); myelocytes, metamyelocytes, and promonocytes (intermediate); band cells, neutrophils, and monocytes (mature). Percentage of cells in each stage of differentiation displayed as an average of 10 fields (400 cells counted), error bars indicate standard deviations. (E) Expression of genes associated with myeloid differentiation was assessed in AML4 primary cells 9 days after transduction by QPCR. Relative fold-difference in expression for miR-150 vs. ECV cells is displayed; error bars represent standard deviations of technical triplicates.

We then examined whether increased miR-150 expression could also promote differentiation of more heterogeneous CD34+ primary patient leukemia samples. For these investigations we transduced 6 AML and 2 myeloid BC CML patient samples with miR-150 or control vectors. Patient characteristics are shown in Table **S1** and included good, intermediate and poor risk AML patients. MiR-150 expression was detectable, but low, in these patient samples with a 150-fold range in expression between samples (Table **S1**). Similar to normal hematopoietic progenitor cells we observed on average a 2 to 3.5-fold increase in CD11b expression in miR-150 expressing cells vs. control cells in 4 of 6 AML samples (one sample t-test, P=0.02, Figure **4A, B**), but no significant change in CD34 expression. The absolute increase in CD11b percent expression in miR-150 cells vs. control cells ranged from 10% to 45%. These changes were also evident on cytospin preparations with increased monocytes and granulocytes in miR-150 expressing cells (Figure **4C, D**). We were unable to grow colonies from the transduced AML patient cells. However, similar to CD34+ progenitor cells from healthy individuals expressing miR-150, we observed an increase in expression of myeloid differentiation associated genes in miR-150 expressing AML cells, with the largest increases observed for S100A8 and S100A9 in AML patient 4 cells (Figure **4E**).

**Figure 5:**
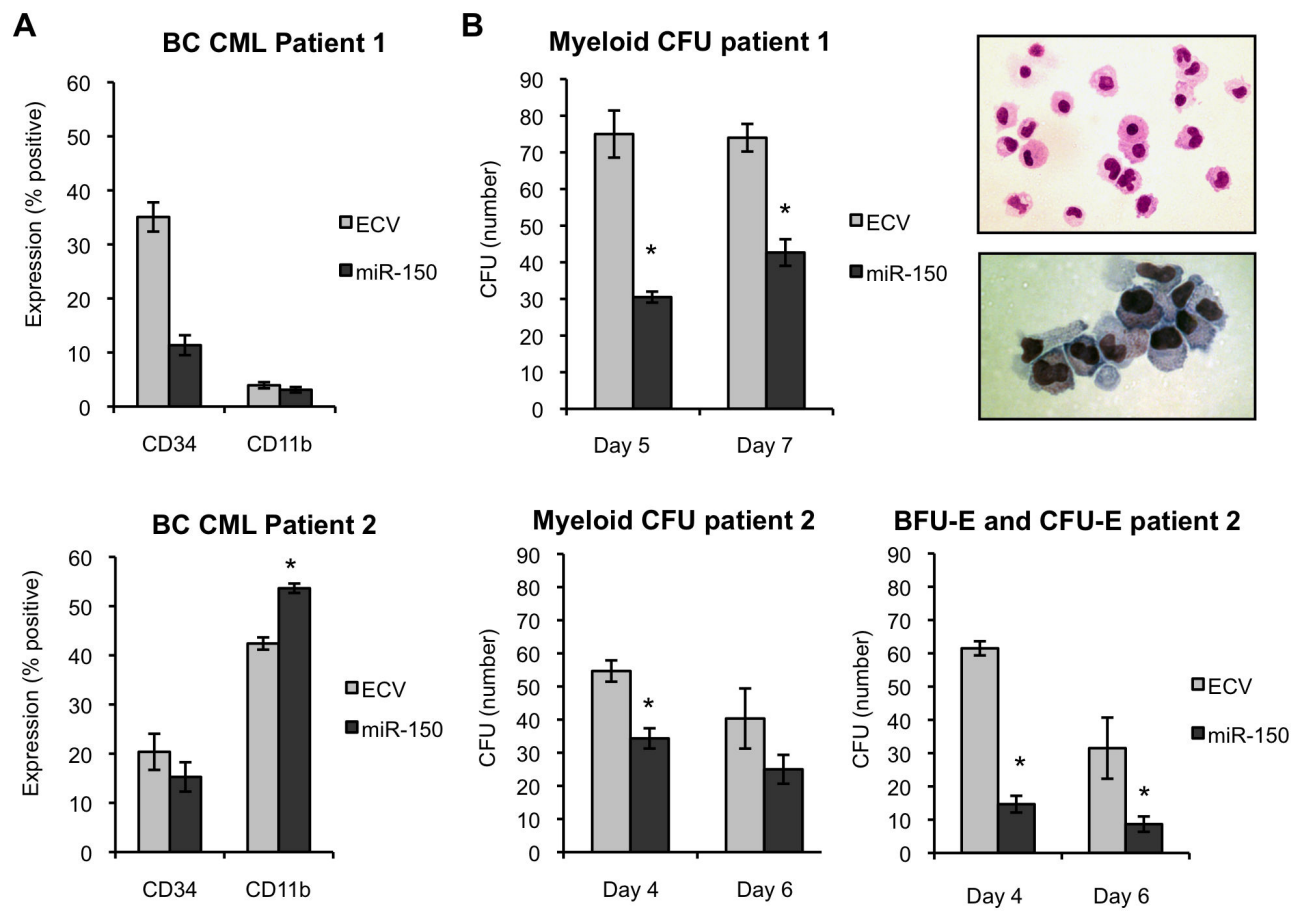
MiR-150 expression promotes myeloid differentiation of primary BC CML patient cells. Primary BC CML patient samples were transduced with pre-miR-150 or empty control lentiviral (ECV) supernatants, sorted for GFP expression, and then assayed by flow cytometry for CD34 and CD11b expression or colony formation by colony-forming unit (CFU) assays. CD34 expression decreases with myeloid differentiation, whereas CD11b expression increases. (A) CD34+ cells from two BC CML patients were assessed for CD34 and CD11b expression 7 days after transduction in the presence of rhSCF, rhIL3, rhIL6, rhGCSF, and rhGMCSF (50 ng/ml each). Data are shown separately for each patient. Duplicates measurements are reported for patient 1 and triplicate measurements for patient 2. (B) BC CML progenitor cells from patient 1 were sorted and plated in triplicate 5 and 7 days after transduction in Methocult™ containing 20% lot-tested FBS, 10% BSA, and cytokines stated above. Colonies were counted 12-16 days after plating. CFU assays demonstrated decreased myeloid CFUs (CFU-M, CFU-G, and CFU-GM combined) in miR-150 versus control primary BC CML patient cells. Individual colonies were plucked, prepared by cytospin, and stained with Wright-Giemsa to validate morphology. A representative example of cells from a CFU-GM colony (from miR-150 expressing BC CML patient 1 cells) shows monocytes, bands, and granulocytes (top photo) and bands and metamyelocytes (bottom photo). BC CML progenitor cells from patient 2 were sorted and plated in triplicate 4 and 6 days after transduction on Methocult™ as above with the addition of EPO (2 U/ml). A similar decrease in myeloid CFUs was observed. Additionally, BFU-E and CFU-E were also decreased in numbers. The mean colony numbers from triplicate plates are shown; error bars indicate standard deviations (*P≤0.*005* for comparisons, Student’s t-test).

For BC CML cases we observed an increase in CD11b in one case (CML2), but no change in CD11b expression in the other case (CML1) (Figure **5A**). CD11b expression was substantially higher in CML2 at the beginning of the experiment. However, overall we did not find an association with baseline CD11b expression and subsequent increase in CD11b expression after miR-150 transduction in the AML patient samples. In one case (CML1) miR-150 as compared to control transduced cells exhibited decreased CD34+ expression similar to progenitors from healthy individuals. In both BC CML patients, miR-150 expression decreased myeloid CFU formation including CFU-GM, CFU-G, and CFU-M. In CML2, we also found that BFU-E and CFU-E formation were decreased in miR-150 expressing cells versus control cells (Figure **5B**). Combined with our observations in normal CD34+ progenitor cells, these observations in AML and BC CML primary patient cells suggest that miR-150 expression promotes differentiation (one sample t-test, P=0.005).

### MiR-150 induced myeloid differentiation is independent of retinoic acid receptor α (RARA) signaling

**Figure 6:**
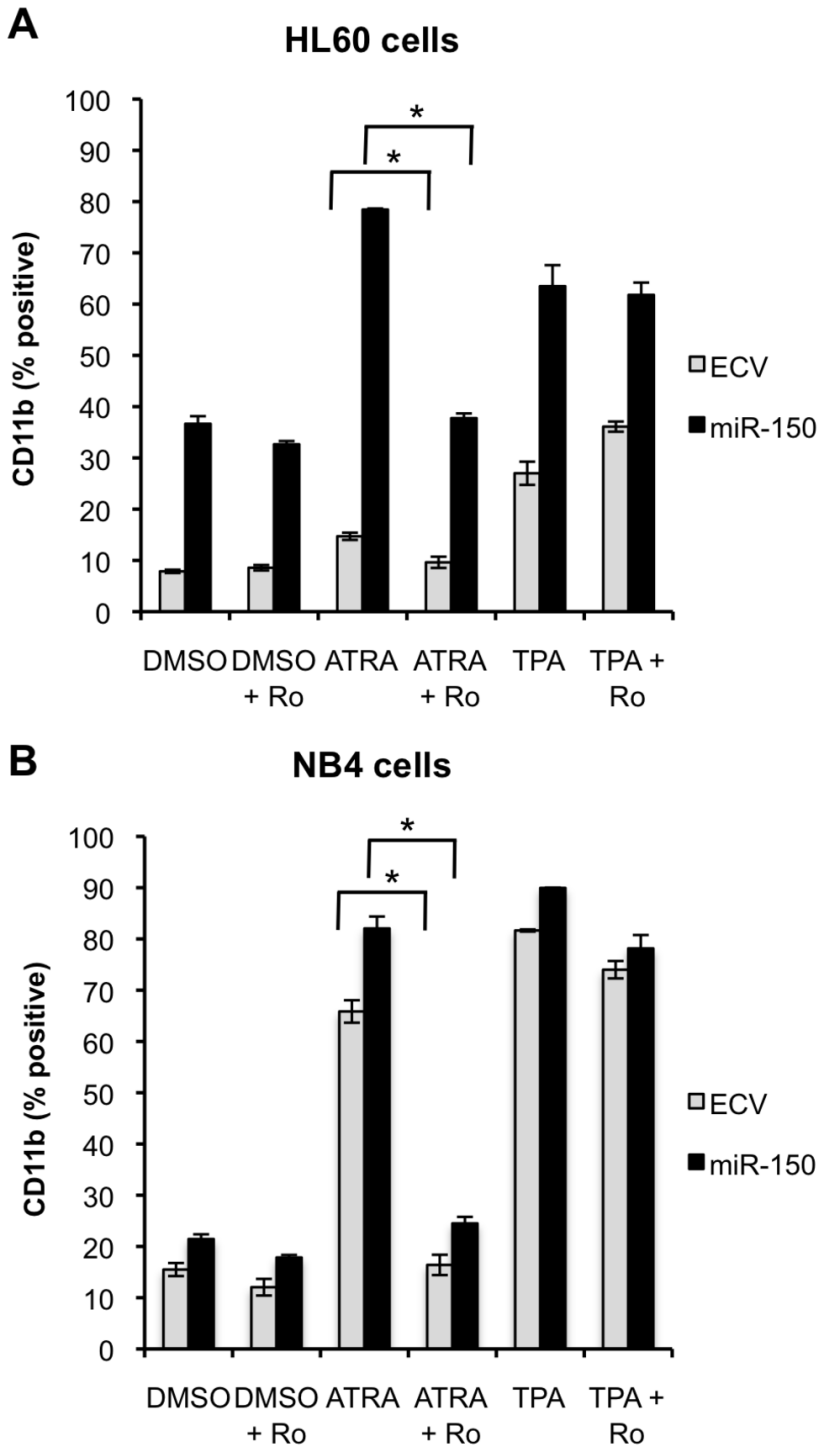
Myeloid differentiation in miR-150 expressing cells is independent of retinoic acid signaling. (A) HL60 or (B) NB4 miR-150 expressing or control (ECV) cells were co-treated with 100 nM selective RAR antagonist (Ro 41-5253) and 1 ng/ml TPA or 0.1 µM ATRA for 48 hours and assessed for CD11b expression by flow cytometry. Ro decreased ATRA induced CD11b expression in both control and miR-150 transduced cells, but did not block induction of CD11b by TPA treatment or by miR-150 expression in the absence of differentiating agent. For HL60 and NB4 cells two independent experiments, each with three technical replicates, were performed for each condition; the means are shown and the error bars represent standard deviations (*P≤0.*05*, Student’s t-test).

To investigate whether miR-150 induced differentiation in the absence of ATRA exposure was also mediated via the retinoic acid receptor (RAR) pathway, RAR signaling was blocked using the selective RARA antagonist Ro 41-5253 (Ro) [42]. MiR-150 expressing or control HL60 and NB4 cells were co-treated with Ro or vehicle control (0.1% DMSO) and TPA or ATRA and assayed for CD11b expression 48 hours after drug exposure. Treatment with Ro had no significant impact on the increase in CD11b expression observed in miR-150 versus control cells after exposure to Ro and vehicle control or TPA (Figure **6**). Control cells exposed to Ro and ATRA exhibited decreased CD11b expression as compared to cells treated with ATRA alone. However, in miR-150 cells Ro only partially reversed the observed increase in CD11b expression. These observations suggest that miR-150 promotes differentiation in the absence of ATRA by pathways distinct from RAR signaling. Supporting this observation, we found that miR-150 expression increased CD11b expression in HL60R cells that are resistant to ATRA as a consequence of a mutation in RARA (data not shown).

### MiR-150 targets MYB to promote myeloid differentiation

To establish how miR-150 promotes myeloid differentiation, we compared the expression of several transcription factors associated with myeloid differentiation in miR-150 versus control transduced cells by QPCR. We observed no consistent differences in gene expression of CEBPA, CEBPB, CEBPD, PU.1, GATA1, and MZF1 (data not shown). In some cases, we observed increased CEPBE expression (Figure **S4**). MYB is a validated target of miR-150 in several cell types and model systems and is highly relevant in leukemogenesis [9,28,29,43]. Consequently, we examined whether miR-150 promotes myeloid differentiation by targeting MYB. We confirmed that miR-150 targets MYB in leukemia cell lines by using a 3’ UTR luciferase reporter assay (Figure **S6**).

**Figure 7:**
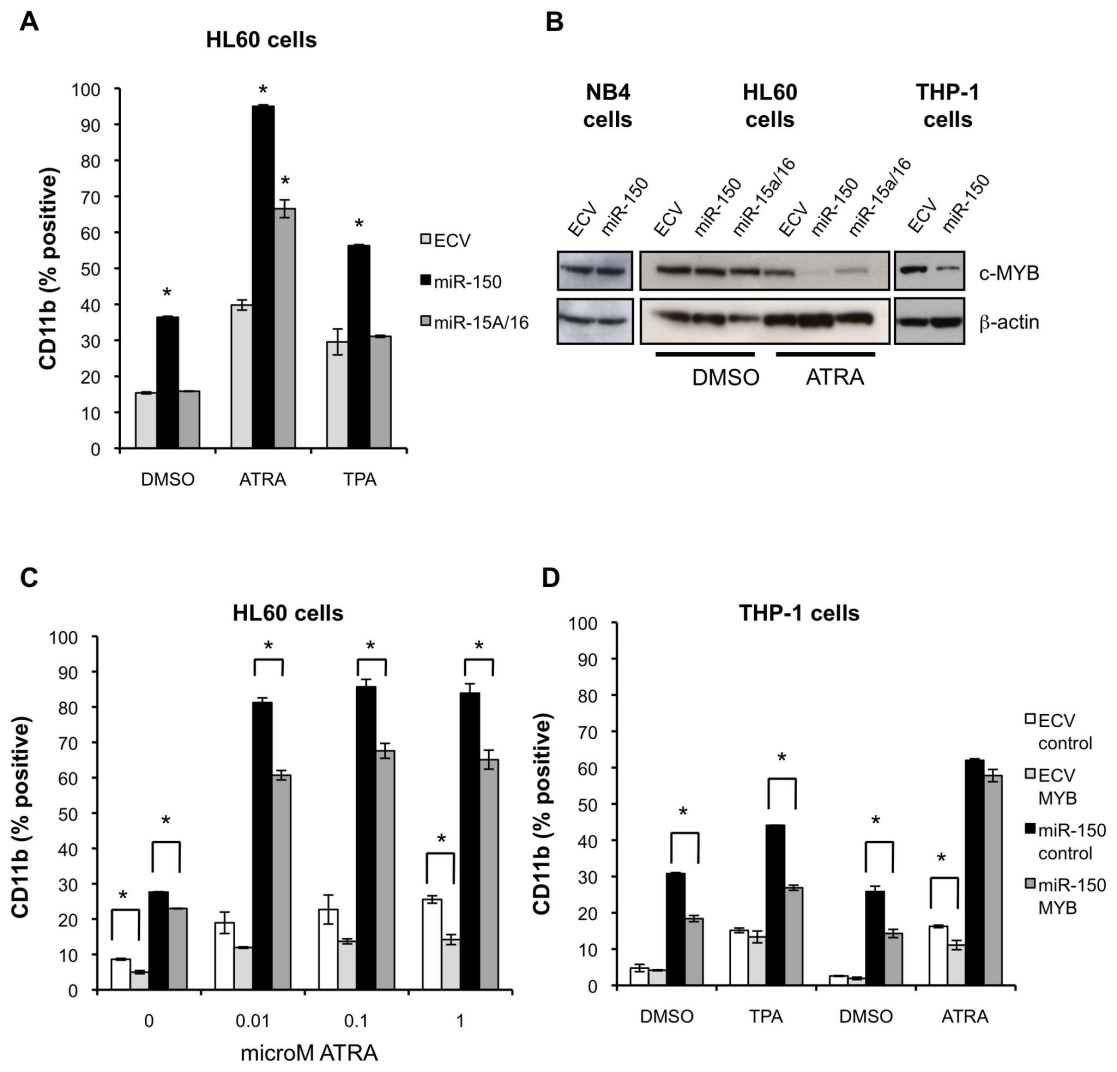
The miR-150 target MYB partially mediates miR-150 induced myeloid differentiation. (A) HL60 cells were transduced with pre-miR-150, pre-miR-15a/miR-16, empty control lentiviral (ECV) supernatants and then treated with vehicle control (0.1% DMSO), ATRA (1 µM) or TPA (1 ng/mL) for 96 hours and assayed for CD11b expression by flow cytometry. MiR-150 increased CD11b expression in all conditions, including in the absence of differentiating agent or with TPA in contrast to miR-15a/miR-16 and ECV cells. (B) MYB protein expression was assayed in NB4, HL60 and THP-1 miR-150 expressing versus control cell lysates by Western blot with the indicated antibodies. Decreased MYB protein expression was most evident in THP-1 cells expressing miR-150 in the absence of differentiating agent. MYB protein was decreased in both miR-150 and miR-15a/miR-16 expressing HL60 cells after 96 hours of ATRA treatment, but was decreased to the greatest extent in miR-150 expressing cells. (C, D) HL60 and THP-1 cells were transduced with miR-150 (GFP-selectable) and MYB∆3’UTR (YFP-selectable, abbreviated MYB) and control vectors and sorted for double positive YFP and GFP cells. MYB∆3’UTR lacks three miR-150 binding sites. (C) HL60 cells were treated with vehicle control (0.1% DMSO) or the indicated concentrations of ATRA for 96 hours and assayed for CD11b expression by flow cytometry. MYB∆3’UTR expression decreased miR-150 induction of CD11b both in the presence or absence of ATRA. (D) THP-1 cells were treated with vehicle control (0.1% DMSO) or TPA (12.5 ng/mL) for 24 hours, or DMSO or ATRA (1 µM) for 48 hours and then assayed for CD11b expression by flow cytometry. MYB∆3’UTR overexpression significantly decreased CD11b expression in miR-150 expressing cells in either the presence or absence of TPA but not ATRA. For A, C, D two independent experiments, each with three technical replicates, were performed for each condition; the means are shown and the error bars represent standard deviations (*P≤0.*05*, Student’s t-test).

We compared our results with miR-150 to miR-15a and miR-16-1, which are encoded by the same primary miRNA transcript and also target MYB [44]. MiR-15a and miR-16-1 expression is increased after ATRA exposure and forced overexpression of these miRNAs enhances ATRA-induced differentiation [45]. In contrast to miR-150, these miRNAs are expressed endogenously at high levels in AML cell lines. In agreement with published reports, lentiviral vector mediated overexpression of miR-15a/16-1 in HL60 cells (3-4 fold overexpression) increased CD11b expression after exposure to ATRA, but did not increase CD11b after exposure to TPA or in the absence of these agents as was seen for miR-150 (Figure **7A**) [45]. MYB protein expression was most decreased in miR-150 expressing THP-1 cells as compared to HL60 or NB4 cells (Figure **7B**). In all cell lines exposed to ATRA MYB was decreased to the greatest extent in miR-150 expressing cells as compared to control cells or miR-15a/16-1 expressing cells (Figure **7B**).

MYB is highly expressed in leukemia, and silencing of MYB has been shown to promote myeloid differentiation, whereas its expression blocks differentiation [33,43,46]. In order to determine more directly if MYB is targeted by miR-150 to promote differentiation in AML cells we expressed MYB that lacks the 3’ UTR (MYB∆3’UTR) containing 3 validated miR-150 binding sites. Thus, when expressed this form of MYB cannot be regulated by miR-150. We co-expressed MYB∆3’UTR from a lentiviral vector also encoding YFP along with miR-150 from a lentiviral vector encoding GFP in HL60 and THP-1 cells [28,29]. Transduced cells were sorted for YFP and GFP expression, treated with ATRA, and analyzed for CD11b expression. MYB∆3’UTR as compared to control transduced HL60 cells exhibited decreased CD11b expression both in presence or absence of ATRA, with the largest differences observed in ATRA treated cells (Figure **7C**). Similar results were seen in THP-1 cells both in the presence or absence of TPA (Figure **7D**). As expected over-expression of this form of MYB inhibited differentiation in all cells, however the effects were most evident in cells expressing miR-150 suggesting that miR-150 downregulation of MYB is partially responsible for miR-150 induced myeloid differentiation.

In order to identify miR-150 targets in addition to MYB that may promote differentiation, we utilized Ingenuity miRNA target filter analysis to identify putative miR-150 targets (extracted from TargetScan) with decreased expression in miR-150 transduced cells as compared to control cells (Table **S4**). In these microarray studies, ATF5 and MYB were among the most statistically significant targets with decreased expression in miR-150 versus control cells in at least one cell line. Additionally QPCR validation studies demonstrated at least a two-fold reduction in expression of IRF8, an important myeloid transcription factor [7], in miR-150 expressing cells vs. control cells in all cell lines examined (Figure **S6A**). However, we could not confirm ATF5 or IRF8 as a direct miR-150 target using 3’ UTR luciferase assays (Figure **S6B**).

## Discussion

Several lines of evidence support the importance of miRNAs in normal and aberrant differentiation. Recently, deletion of *Dicer1*, which is an enzyme that is essential for miRNA biogenesis, in murine myeloid committed progenitors provided evidence that a miRNA-controlled switch may be critical in normal myeloid differentiation [47]. In this work we have identified a new role for miR-150 in promoting myeloid differentiation of AML cell lines, CD34+ progenitor cells from healthy individuals, and primary BC CML and AML patient cells. Although other studies have identified miRNA expression changes with ATRA-induced differentiation, in many cases it remains unclear if differential expression of these miRNAs contributes to altered differentiation or is a consequence of ATRA-induced differentiation [45,48,49]. Few studies have identified miRNAs that alter differentiation in the absence of these agents [20-23]. Our studies suggest that miR-150 plays a direct role.

Understanding the function, regulation, and targets of specific miRNAs has proven difficult and remains a main bottleneck in understanding the contributions of miRNAs to normal and malignant hematopoiesis. Recent studies highlight our growing understanding of the effects of altered miR-150 expression. MiR-150 has been shown to play a role in normal lymphoid and megakaryocyte development, but no role has yet been described in myelopoiesis [9,28]. The clinical relevance of decreased miR-150 expression has also been highlighted by studies describing its role as a tumor suppressor in lymphoma and most recently examining how decreased expression promotes leukemogenesis in MLL-AF9 and MLL-ENL rearranged leukemia [27,31]. This last study focused on decreased proliferation as a consequence of miR-150 expression, but our work suggests loss of miR-150 expression also inhibits myeloid differentiation, a key characteristic of acute leukemia. We have found that miR-150 expression increases with terminal myeloid differentiation and increased expression of miR-150 promotes granulocytic and monocytic differentiation of CD34+ progenitor cells from healthy individuals. These observations suggest that miR-150 plays a role in normal myelopoiesis. In AML cell lines and primary human AML and BC CML progenitors with various cytogenetic and molecular abnormalities, including normal karyotype AML, we observed that miR-150 expression promotes myeloid differentiation. Our observations extend the relevance of decreased miR-150 expression to other leukemia subtypes and reveal another important function of miR-150 that is lost in AML and BC CML.

MiR-150 regulation of its direct target MYB is partly responsible for the myeloid differentiation phenotype observed in leukemia cell lines. Our data show that overexpression of MYB, without its known miR-150 binding sites in the 3’ UTR, decreases CD11b expression. These results are consistent with published reports that MYB expression blocks terminal differentiation and MYB silencing enhances monocyte/macrophage differentiation [32,33]. There are several possible explanations for the observation of only a partial reversal of differentiation. There is another putative miR-150 binding site in the coding region of MYB that is included in our MYB∆3’UTR construct, and may allow some modulation of MYB by miR-150 [28,29]. Alternatively, it is likely that additional miR-150 targets contribute to differentiation. We used gene expression profiling and Ingenuity miRNA target filter analysis to help prioritize predicted direct targets of miR-150. ATF5 and IRF8, two predicted targets with decreased expression, do not appear to be targeted directly by miR-150. Our studies suggest that regulation of mature miR-150 and not miR-150* targets promotes myeloid differentiation, and we continue to examine other promising direct and indirect targets of miR-150. Additionally, our data suggest that miR-150 may cooperate with other miRNAs to promote differentiation. Our miRNA expression studies of PL21 cells identified that miR-150 expression increased miR-223 expression, a miRNA associated with myeloid differentiation [16].

Analysis of our gene expression data in three AML cell lines identified activation of transcription factors and enrichment of pathways associated with myeloid differentiation in miR-150 expressing cells (Table **S3, S5**). Ingenuity Pathways Analysis identifies putative upstream regulators that are predicted to be activated or inhibited in these expression data. Although not differentially expressed in our experiments, the top statistically significant transcription factor predicted to be activated in miR-150 expressing cells in all cell lines was CEBPA (HL60, *P*=5.87E-9; PL21, *P*=5.77E-10; and THP-1, *P*=2.20E-11). CEBPA plays an established role in granulocytic differentiation and is dysregulated in AML and BC CML [50,51]. Other top candidates included cytokines, growth factors and transcription factors associated with myeloid differentiation including CEBPE, IL1Β, and GM-CSF. CEBPE has been implicated in terminal myeloid differentiation [52,53]. CEBPA along with G-CSF, GM-CSF, IL3, and IL6 play a role in steady-state granulopoiesis, whereas IL1Β also appears to play a role in reactive granulopoiesis [54]. These analyses also identified another enriched pathway that may participate in myeloid differentiation in miR-150 expressing cells, PPARG. Recent studies suggest PPARG agonists induce differentiation, in addition to growth arrest and apoptosis, in AML cells and augment the effects of ATRA [55]. Given our observation that a RAR antagonist did not reverse the myeloid differentiation phenotype observed and only partly reversed the phenotype in cells treated with ATRA, it is tempting to speculate that signaling through PPARG/RXR or activation of CEBPA or CEBPE through mechanisms yet to be established may play a role in differentiation of miR-150 expressing cells [55].

Given the importance of miR-150 not only in myeloid differentiation, but also in megakaryocytic, lymphoid, and natural killer cell development, and most recently in adhesion of bone marrow progenitors, elucidating how miR-150 is regulated is also highly significant [9,28-30,56]. In MLL-AF9 AML the MYC/LIN28 axis inhibits post-transcriptional maturation of miR-150 [27]. MYC regulation of miR-150 may be a common mechanism of its repression more broadly in AML. In our pediatric cohort, however, we did not find a correlation between MYC or LIN28 expression and miR-150 expression, but MYC was moderately to highly expressed in most samples as has been previously described [57]. Using TRANSFAC analysis we have identified several transcription factor binding sites, including MYB, STAT5A, IKZF1, MZF1, SP1 and LMO2, in the miR-150 putative promoter region. MYB and STAT5A expression are enhanced by BCR-ABL expression and both are essential for BCR-ABL mediated transformation [58,59]. LMO2 is important in murine hematopoiesis and the inverse relationship between miR-150 and LMO2 expression in published reports suggests LMO2 may repress miR-150 expression in B and T cell and erythroid/megakaryocytic differentiation [32,60,61].

In conclusion, we have demonstrated that miR-150 promotes myeloid differentiation of normal human progenitor cells and AML and BC CML progenitor cells. Understanding how differentiation is blocked and how this block can be overcome in leukemia has major clinical significance. For example, differentiation therapy in acute promyelocytic leukemia (APL) has resulted in long-term remissions without the need for conventional chemotherapy [62,63]. Unfortunately, strategies to differentiate other types of AML are limited by a lack of understanding of the critical pathways controlling differentiation in leukemia cells. A broader understanding of miRNA regulation and mechanisms contributing to blocked differentiation, particularly for miR-150, may provide a new path towards the goal of effective and non-toxic differentiation therapy.

## Materials and Methods

### Ethics Statement

In accordance with the Declaration of Helsinki, all patients (or guardians on behalf of the pediatric patients) provided written informed consent for the collection and use of their biospecimens for research purposes under studies approved by the British Columbia Cancer Agency Research Ethics Board (Vancouver, BC, Canada) and the Fred Hutchinson Cancer Research Center Institutional Review Board (FHCRC; Seattle, WA, USA). Clinical data were de-identified in compliance with Health Insurance Portability and Accountability Act regulations.

### Plasmid Constructs

Pre-miRNAs were expressed using a lentiviral vector, pLMF-MSCV-GFP, under the control of a MSCV promoter with GFP expression controlled by a phosphoglycerate kinase (hPGK) promoter [35]. The genomic regions surrounding the pre-miR-150 sequence were amplified using primers 5’-GGGCCGCGGGGAGTGGGTGTGCAGTTTCT-3’ and 5’-GGGCGTACGACTTTGCGCATCACACAGAG-3’. Mutations were created using the Quikchange XL Kit (Agilent Technologies, Inc., Santa Clara, CA, USA). The genomic regions surrounding the pre-miR-15a/miR-16-1 sequences were amplified using the primers 5’-GGGCCGCGGATTCTTTAGGCGCGAATGTG -3’ and 5’-GGGCGTACGTTGATGGCATTCAATACAATTATTA -3’. MYB∆3’UTR was cloned into pLMF-MSCV-YFP from a cDNA clone (OriGene, Rockville, MD, USA).

### Primary patient cell and cell line cultures

Adult and pediatric patient samples were obtained from the Leukemia Repository at the Fred Hutchinson Cancer Research Center and from Children’s Oncology Group. PL21 [64], KG1 [65], MV4-11 [66], K562 [67], LAMA84 [68], and Kcl-22 [69] cells were cultured in RPMI 1640 (Gibco-Invitrogen, Carlsbad, CA, USA) supplemented with 10% fetal bovine serum (FBS) (JR Scientific, Inc, Woodland, CA, USA), 1% penicillin/streptomycin, and 5% L-glutamine (Gibco-Invitrogen). HL60 [70], NB4 [71], and THP-1 [72] cells were cultured as above except with 10% cosmic calf serum (Hyclone, Logan, UT, USA). Normal CD34+ PBSCs and primary BC CML and AML patient cells were cultured in IMDM (Gibco-Invitrogen) containing 20% lot-tested FBS (Hylclone), 1% penicillin/streptomycin, 2mM glutamine, 0.1 mM β-mercaptoethanol, 50 ng/ml each of rhSCF, rhIL3, rhIL6, GCSF, and GMCSF (Peprotech, Rocky Hill, NJ, USA), and 2 U/mL EPO (PROCRIT®, Janssen Biotech, Inc., Horsham, PA, USA). Methods for primary cell culture and transduction are described in detail in Methods **S1** [35]. Cell proliferation was measured by the ATPlite assay (PerkinElmer, Waltham, MA, USA). TPA, ATRA (Sigma-Aldrich, St. Louis, MO, USA), and Ro 41-5253 (Enzo Life Sciences, Farmingdale, NY, USA) were resuspended according to manufacturer’s instructions in DMSO. Wright-Giemsa stained myeloid cells were characterized into 3 groups as follows: 1) immature blast cells with scant cytoplasm; 2) intermediate stage cells with increased cytoplasm-to-nucleus ratio, more irregularly shaped or indented nuclei; and 3) mature cells, which include monocytes with increased vacuoles and smaller round or kidney bean shaped nuclei and granulocytes or bands with lobulated nuclei [73]. Images of Wright-Giemsa--stained cytospin preparations were obtained with a Nikon Eclipse E800 microscope with 20X and 40X objectives, Olympus digital camera, and MagnaFire software.

### Flow cytometry

Flow cytometry and cell sorting were performed on FACSCanto1 and FACS Aria (Becton Dickinson Immunocytometry Systems (BD), Franklin Lakes, NJ, USA). Cell surface marker staining was performed in 1XPBS and 0.1% BSA or 1% FBS with the following antibodies: APC CD11b, PE CD34, PE CD14, 7AAD, and appropriate isotype controls (BD). FlowJo (TreeStar Inc.) was used for analysis. Positivity for 7AAD was used to exclude nonviable cells, and thresholds for positivity were determined using unstained cells and isotype control antibodies.

### CFU assays

Transduced BC CML, AML, and CD34+ PBSCs from healthy individuals were plated in triplicate in MethoCult™ (STEMCELL Technologies Inc., Vancouver, Canada) supplemented with 20% lot-tested FBS, 10% BSA (Biocell, Laboratories, Inc., Rancho Dominguez, CA) and cytokines as described above and then grown in a hypoxic incubator (3% O_2_). CFU colonies were counted 14 to 16 days after plating [35].

### Statistical methods

To measure miR-150 expression in patient samples, technical replicates were performed for each patient sample and a Student’s t-test was used to determine p-values for comparisons between groups. For cell line experiments proliferation and cell surface marker expression were compared across experimental conditions. Biological replicates of cell line experiments, each performed in duplicate or triplicate (as technical replicates) for each condition, were displayed as mean values and treated as independent data to assign p-values using a Student’s t-test. Error bars represent the standard deviation of measurements. For the primary patient cell experiments we compared cell surface marker expression or CFU formation between experimental and control conditions over time. Error bars represent the standard deviation of measurements performed as technical replicates in duplicate or triplicate for each patient sample. For CFU assays a Student’s t-test was used for comparisons. A one sample t-test was used to determine whether CD11b expression in AML experiments or across all experiments (AML, BC CML, and healthy patient progenitor cells) was different in miR-150 expressing cells vs. control cells.

### Protein extraction and Western blots

Cell pellets were lysed in RIPA buffer containing 1X complete protease inhibitor (PI) cocktail (Roche, Mannheim, Germany) or for HL60 cells in 1% Triton X-100, 0.2% SDS, and 7X PI, 1 mM N-ethylmaleimide, and 0.3 mM di-isopropyl-fluorophosphate (Sigma). Protein concentrations were quantified with DC Protein Assay (Bio-Rad, Hercules, CA, USA) and 30 µg of whole-cell lysates were separated by SDS–PAGE. Blots were probed using anti-MYB (EMD Millipore, Billerica, MA, USA) and anti-β-actin (Sigma) antibodies.

### RNA isolation and quantitative RT-PCR

Total RNA was isolated with the miRNeasy kit (Qiagen, Valencia, CA, USA) or by the Trizol method (Life Technologies, Carlsbad, CA, USA). CD34+ sorted and unsorted NBM cells were from Lonza Inc. (Allendale, NJ, USA). Gene expression was analyzed by Taqman mRNA (Applied Biosystems (ABI), Life Technologies, Grand Island, NY, USA), PrimeTime QPCR Assays (Integrated DNA Technologies, Inc, Coralville, IA, USA) or MicroRNA Assays (ABI). Relative expression is reported as fold change (ΔC_t_ and ΔΔC_t_ calculations) normalized to GUSB for mRNA expression and RNU49 for miRNA expression. All samples were run in duplicate.

### Microarray analysis

Files for raw and normalized data have been deposited in the GEO public database under accession number GSE40147 and are available at the following link: http://www.ncbi.nlm.nih.gov/geo/query/acc.cgi?token=ftyrxqmkyeoukhi&acc=GSE40147 Ingenuity Pathways Analysis (Ingenuity Systems®, www.ingenuity.com) was used to determine biologically enriched pathways, functions, miRNA targets, and upstream regulators (Methods **S1**).

### MiRNA sequencing

Pediatric AML patient RNA was processed and sequenced using Illumina Hiseq 2000 (San Diego, CA, USA). Raw data was normalized and analyzed using Bioconductor (Methods **S1**).

## Supporting Information

Figure S1 **MiR-150 expression increases during terminal myeloid differentiation.** (A) MiR-150 expression data was obtained and analyzed from Petriv et al. for various stem (dark blue), progenitor (medium blue) and mature (light blue) myeloid populations isolated from healthy mice by flow cytometry using cell surface markers as described [10]. MiR-150 expression is displayed as log_2_ molecule counts converted from Ct values. Hematopoietic stem cells (HSC) with high self-renewal capacity CD150^+^ (ES 150+), HSC low self-renewal capacity CD150^-^ (ES 150-), HSC CD150^+^ CD48^-^ (SLAM), Lin^-^ sca-1^+^ ckit^+^ (LSK), granulocyte/macrophage progenitor (GMP), common myeloid progenitor (CMP). (B) MiR-150 expression increases in healthy human CD34+ PBMCs during differentiation in liquid culture in the presence of GM-CSF and G-CSF as determined by QPCR at the indicated days in culture. Expression in each sample is normalized to expression in that sample on day 10 in culture.
(TIF)

Figure S2 **MiR-150 expression is similar to normal BM in miR-150 transduced cell lines and primary cells.** (A) Expression levels of mature miR-150 and miR-150*, which have very low expression in these cell lines at baseline, are shown in pre-miR-150 transduced NB4, HL60, PL21, and THP-1 cells. Expression levels are similar to NBM (n=5) as measured by QPCR. Expression is shown as fold-change relative to miR-150 expression in NBM on a log_10_ scale. Three independent experiments, each with two technical replicates were performed; the means are shown and the error bars represent standard deviations. (B) Mature miR-150 expression increased to levels similar to normal NBM in miR-150 transduced primary human normal CD34+ PBSC and human primary leukemia patient samples compared to control transduced cells. Two technical replicates were performed; the means are shown.
(TIF)

Figure S3 **MiR-150 expression decreased proliferation in HL60 and NB4 cells compared to control cells.** (A) HL60 cells and (B) NB4 cells were transduced with miR-150 or empty control (ECV) lentivirus, sorted for GFP, and 8 days post transduction plated in triplicate with the indicated concentrations of ATRA or vehicle control (0.1% DMSO). Proliferation was measured by ATPlite assay at the indicated hours post treatment. Three independent experiments, each with two technical replicates were performed; the means are shown and the error bars represent standard deviations.
(TIF)

Figure S4 **MiR-150 expression induces myeloid differentiation gene expression in AML cell lines.** Expression of genes associated with myeloid differentiation [4] was assessed by QPCR in PL21, HL60 and THP-1 cells transduced with miR-150 versus empty control virus (ECV) 7-9 days after transduction. Relative fold-difference in expression for miR-150 vs. ECV cells is displayed; error bars represent standard deviations of biological triplicates. S100A8 and S200A9 are shown in a separate figure due to scale of expression differences for these genes.
(TIF)

Figure S5 **Myeloid differentiation in miR-150 expressing cells is mediated through the mature miR-150 strand.** (A) Seed sequences of miR-150 mature or star strand were mutated in the pre-miR-150 expression vector as indicated. (B, D) HL60 cells and (C, E) NB4 cells were transduced with the various pre-miR-150 lentiviral vector constructs or empty control (ECV) lentivirus. Both mature miR-150 and miR-150* strands are expressed in pre-miR-150 expressing cells as measured by QPCR. Mutation of either miR-150 mature or star seed sequence resulted in absent or low expression of the mutated strand, but maintained expression of the opposite strand, although at a 10-fold decrease relative to the unmutated pre-miR-150 construct. (D) Transduced HL60 and (E) NB4 cells were assayed for CD11b expression by flow cytometry. Mutations in the mature miR-150 strand but not miR-150* abrogated miR-150 induced CD11b expression, indicating mature miR-150 targets mediate the differentiation phenotype. Two independent experiments, each with three technical replicates were performed; the means are shown and the error bars represent standard deviations (*P <0.05, Student’s t-test).
(TIF)

Figure S6 **MiR-150 directly regulates the putative target MYB.** (A) Expression of putative miR-150 targets, MYB, ATF5 and IRF8, was examined by QPCR in PL21, HL60 and THP-1 cells transduced with miR-150 versus empty control virus (ECV) 7-9 days after transduction. The relative fold-difference in expression for miR-150 vs. ECV cells is displayed. Three independent experiments, each with two technical replicates were performed; the means are shown and the error bars represent standard deviations No difference in expression between miR-150 vs. ECV cells is represented as 1. (B) K562 cells were co-transfected with 3'UTR LightSwitch *Renilla* luciferase reporters and pre-miR-150 or pre-miR-150 double mutant expression plasmids, and pGL3-Promoter *Firefly* luciferase as transfection control. GAPDH 3'UTR, random genomic sequence (RO3) 3'UTR, and empty vector served as negative controls. Relative luciferase units (RLU) represent *Renilla*/*Firefly* luminescence reads of the ratio of miR-150 vs. the double mutant miR-150 construct. Three independent experiments, each with three technical replicates were performed; the means are shown and the error bars represent standard deviations (*P≤0.005, Student’s t-test).
(TIF)

Methods S1 (DOCX)

Table S1 **Adult AML and BC CML patient characteristics with miR-150 relative expression.** Descriptions of patient samples with disease status and cytogenetics listed where applicable. MiR-150 expression levels in each sample are shown relative to average normal bone marrow (NBM) miR-150 expression as determined by QPCR (used for Figure **1**). Highlighted samples were used for functional assays in Figure **4** and Figure **5**. ND is not determined or documented; NN is normal karyotype; PB is peripheral blood, BM is bone marrow.
(XLS)

Table S2 **Pediatric AML patient characteristics and statistical analysis of miR-150 expression in samples by cytogenetic and mutational status.** (S2A) Descriptions of pediatric AML samples with disease status and cytogenetic and molecular abnormalities listed where applicable, and miR-150 expression levels determined by RNA sequencing (log_2_ scale, see Methods) (S2B). Statistical analysis of mean miR-150 expression (log_2_ scale) for pediatric AML classified by various cytogenetic (t(8;21), t(9.11), inversion 16 (inv16), trisomy 8 (tri8)), mutation (FLT3 ITD, NPM1, NPM1 FLT3 negative, CEBPA, WT1), or prognostic risk status (good vs. high and standard vs. high).
(XLS)

Table S3 **Top statistically significant gene expression changes concordant in PL21, HL60, and THP1 cells.** Top statistically significant gene expression changes are displayed as concordant in PL21, HL60, and THP1 cells (S3A) or concordant in PL21 and THP1 cells (S3B). The z-score refers to the statistical significance of the difference in expression in either PL21 cells or HL60 cells. Green text represents decreased expression in miR-150 cells as compared to control cells and red text represents increased expression.
(XLS)

Table S4 **Top statistically significant putative miR-150 targets decreased in PL21, HL60, and THP1 cells.** Top statistically significant putative miR-150 targets with the largest decreases in gene expression sorted by statistical significance in PL21 cells (S4A), HL60 cells (S4B), THP1 cells (S3C), and all 3 cell lines (combined analysis, S4D). The source of the target prediction is shown in addition to the confidence level associated with this prediction. The z-score refers to the statistical significance of decreased gene expression in the array data in miR-150 cells as compared to control cells.
(XLS)

Table S5 **IPA Upstream Regulator Analysis was used to identify putative upstream regulators that were activated (or inhibited) in the gene expression data for PL21, HL60, and THP1 cells.** IPA Upstream Regulator Analysis was used to identify putative upstream regulators that were activated (or inhibited) in the gene expression data for PL21, HL60, and THP1 cells in miR-150 cells as compared to control cells. Additionally candidates that were statistically significantly enriched even if direction could not be ascertained were also identified. The P-value is calculated using Fisher’s Exact Test. Upstream regulators include transcription factors, cytokines, miRNAs, receptors, kinases, and chemicals and drugs. Activation or inhibition is assigned based on relationships derived from the literature and various databases and the direction of expression change in the array data provided.
(XLS)
