# Supplementary figures and images for: MicroRNA-150 Expression Induces Myeloid Differentiation of Human Acute Leukemia Cells and Normal Hematopoietic Progenitors
Source: PLoS One. 2013 Sep 24;8(9):e75815. doi: 10.1371/journal.pone.0075815 (PMC3782459; doi:10.1371/journal.pone.0075815)

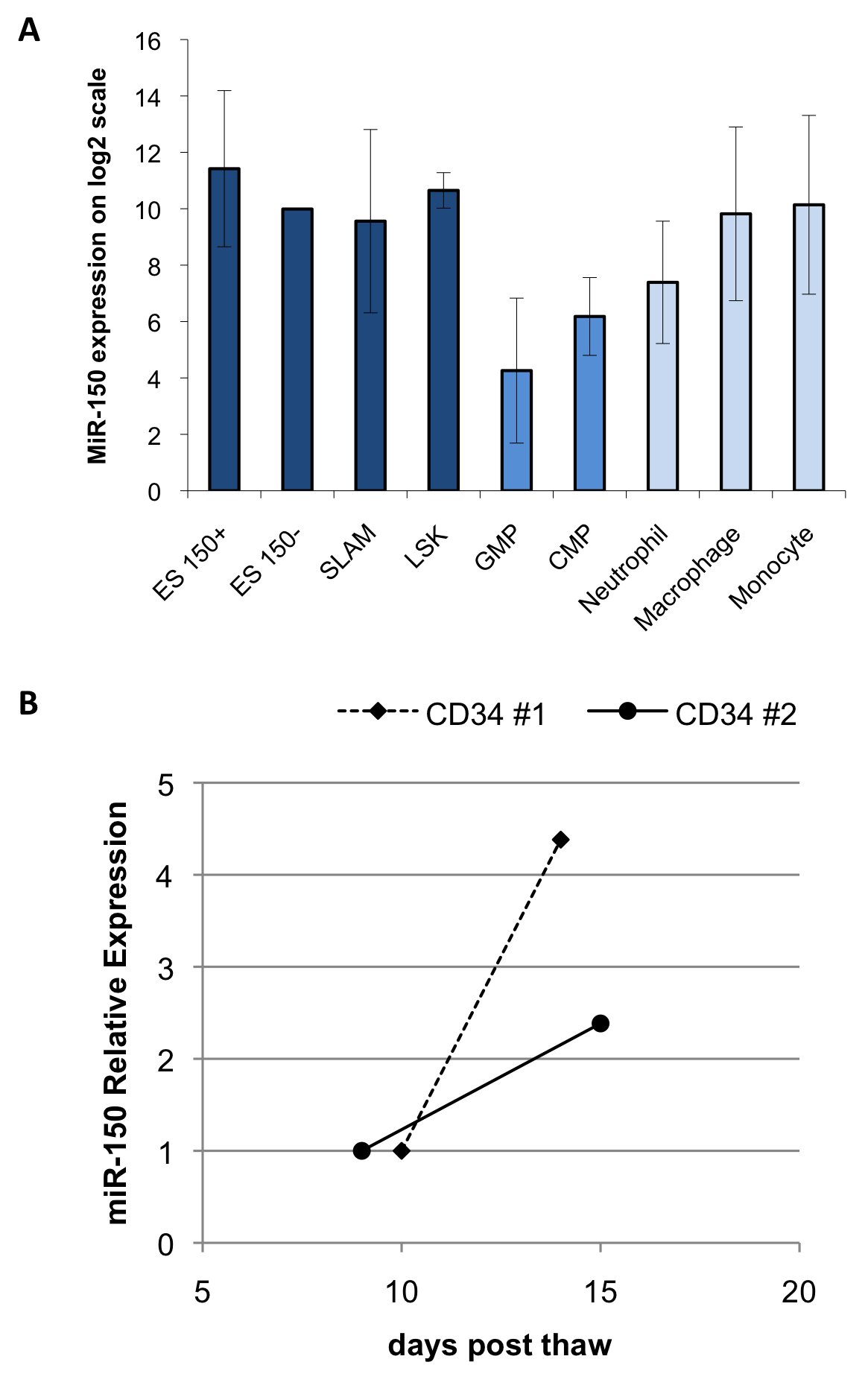

Supplement: Figure S1 — MiR-150 expression increases during terminal myeloid differentiation. (A) MiR-150 expression data was obtained and analyzed from Petriv et al. for various stem (dark blue), progenitor (medium blue) and mature (light blue) myeloid populations isolated from healthy mice by flow cytometry using cell surface markers as described [10]. MiR-150 expression is displayed as log2 molecule counts converted from Ct values. Hematopoietic stem cells (HSC) with high self-renewal capacity CD150+ (ES 150+), HSC low self-renewal capacity CD150- (ES 150-), HSC CD150+ CD48- (SLAM), Lin- sca-1+ ckit+ (LSK), granulocyte/macrophage progenitor (GMP), common myeloid progenitor (CMP). (B) MiR-150 expression increases in healthy human CD34+ PBMCs during differentiation in liquid culture in the presence of GM-CSF and G-CSF as determined by QPCR at the indicated days in culture. Expression in each sample is normalized to expression in that sample on day 10 in culture. (TIF) [file pone.0075815.s001.tif]

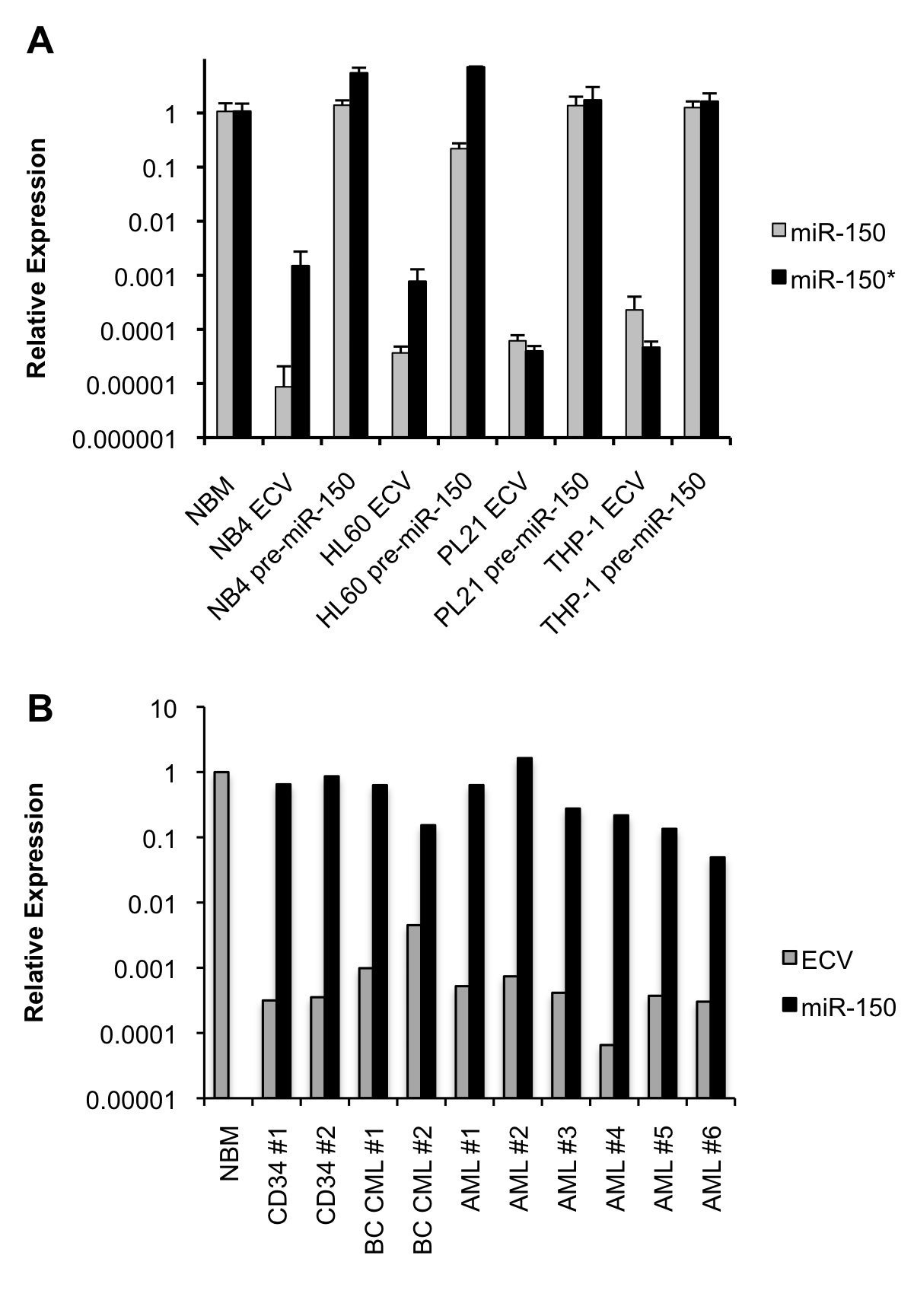

Supplement: Figure S2 — MiR-150 expression is similar to normal BM in miR-150 transduced cell lines and primary cells. (A) Expression levels of mature miR-150 and miR-150*, which have very low expression in these cell lines at baseline, are shown in pre-miR-150 transduced NB4, HL60, PL21, and THP-1 cells. Expression levels are similar to NBM (n=5) as measured by QPCR. Expression is shown as fold-change relative to miR-150 expression in NBM on a log10 scale. Three independent experiments, each with two technical replicates were performed; the means are shown and the error bars represent standard deviations. (B) Mature miR-150 expression increased to levels similar to normal NBM in miR-150 transduced primary human normal CD34+ PBSC and human primary leukemia patient samples compared to control transduced cells. Two technical replicates were performed; the means are shown. (TIF) [file pone.0075815.s002.tif]

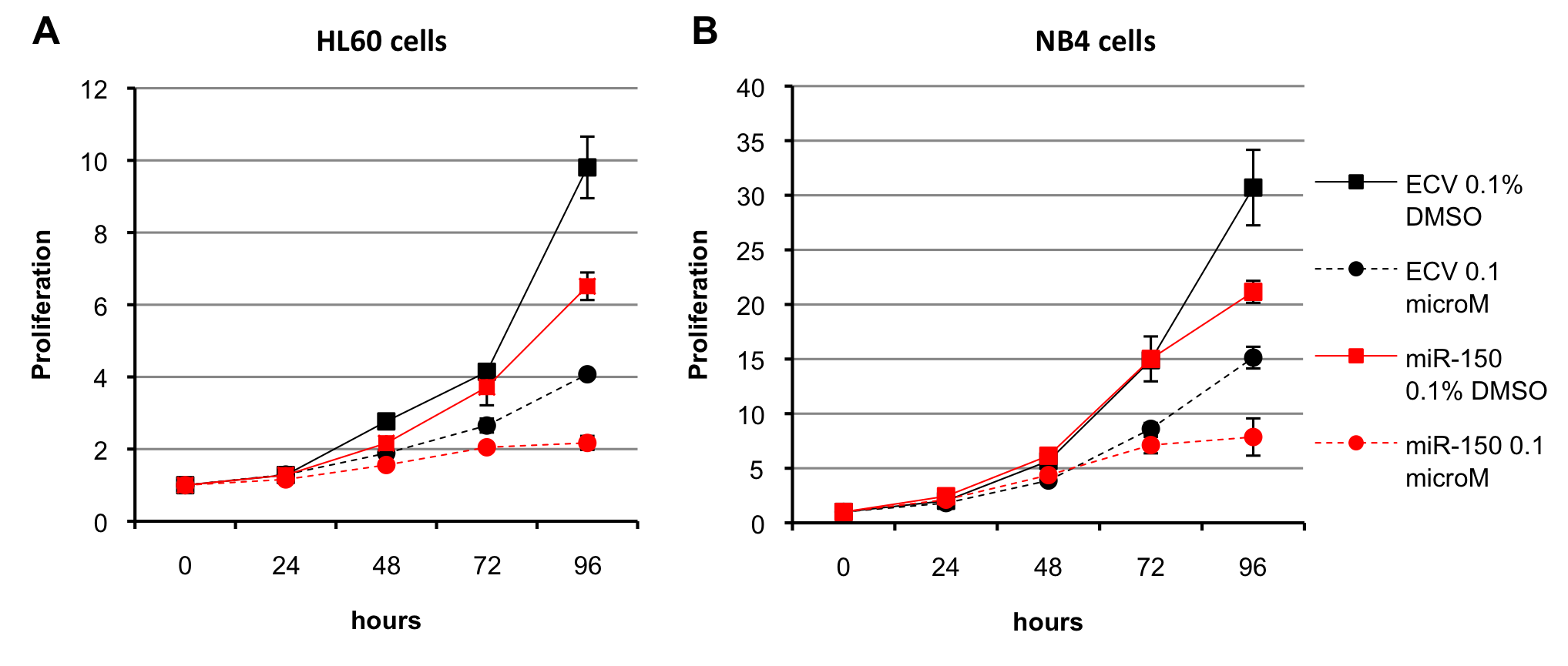

Supplement: Figure S3 — MiR-150 expression decreased proliferation in HL60 and NB4 cells compared to control cells. (A) HL60 cells and (B) NB4 cells were transduced with miR-150 or empty control (ECV) lentivirus, sorted for GFP, and 8 days post transduction plated in triplicate with the indicated concentrations of ATRA or vehicle control (0.1% DMSO). Proliferation was measured by ATPlite assay at the indicated hours post treatment. Three independent experiments, each with two technical replicates were performed; the means are shown and the error bars represent standard deviations. (TIF) [file pone.0075815.s003.tif]

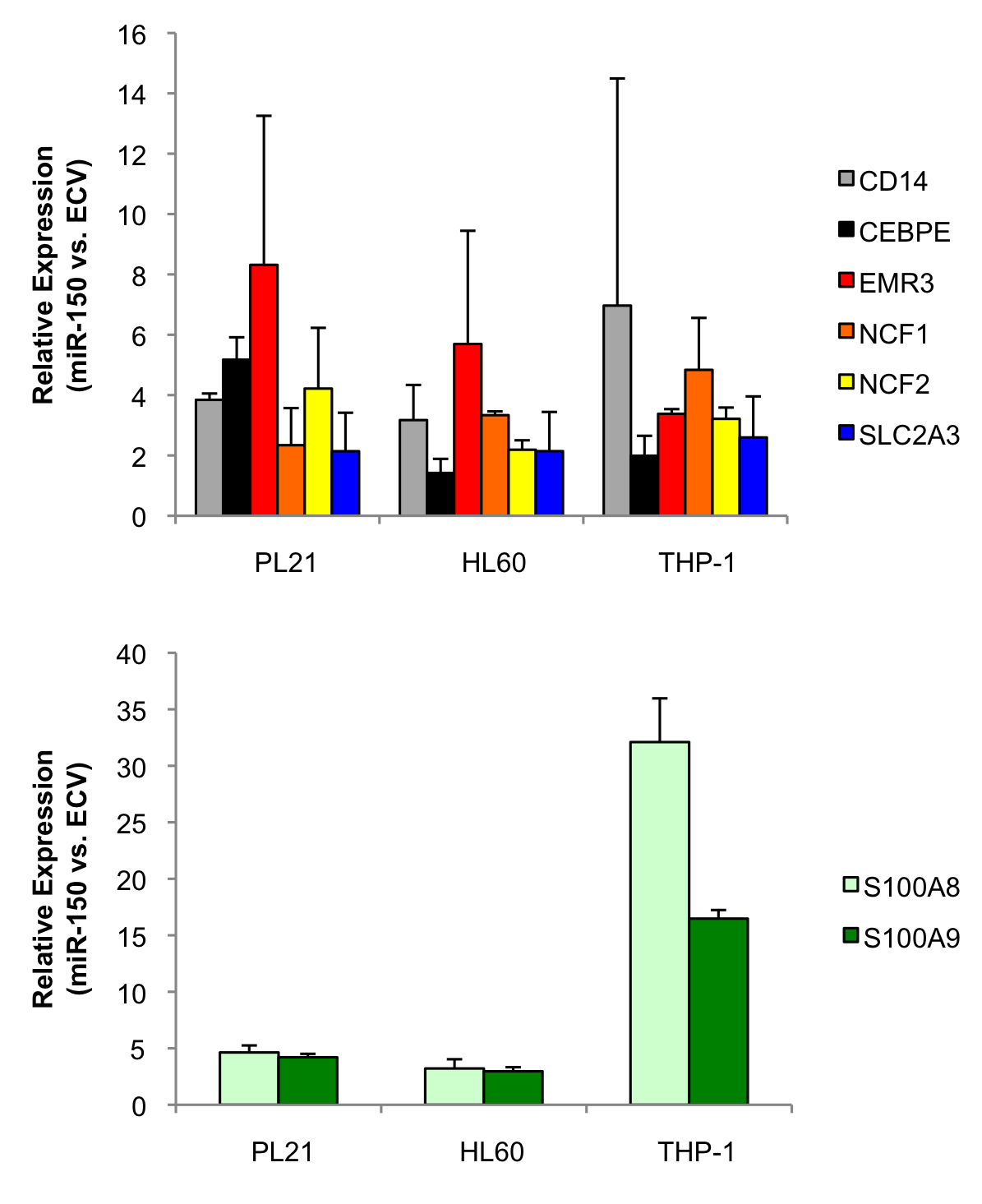

Supplement: Figure S4 — MiR-150 expression induces myeloid differentiation gene expression in AML cell lines. Expression of genes associated with myeloid differentiation [4] was assessed by QPCR in PL21, HL60 and THP-1 cells transduced with miR-150 versus empty control virus (ECV) 7-9 days after transduction. Relative fold-difference in expression for miR-150 vs. ECV cells is displayed; error bars represent standard deviations of biological triplicates. S100A8 and S200A9 are shown in a separate figure due to scale of expression differences for these genes. (TIF) [file pone.0075815.s004.tif]

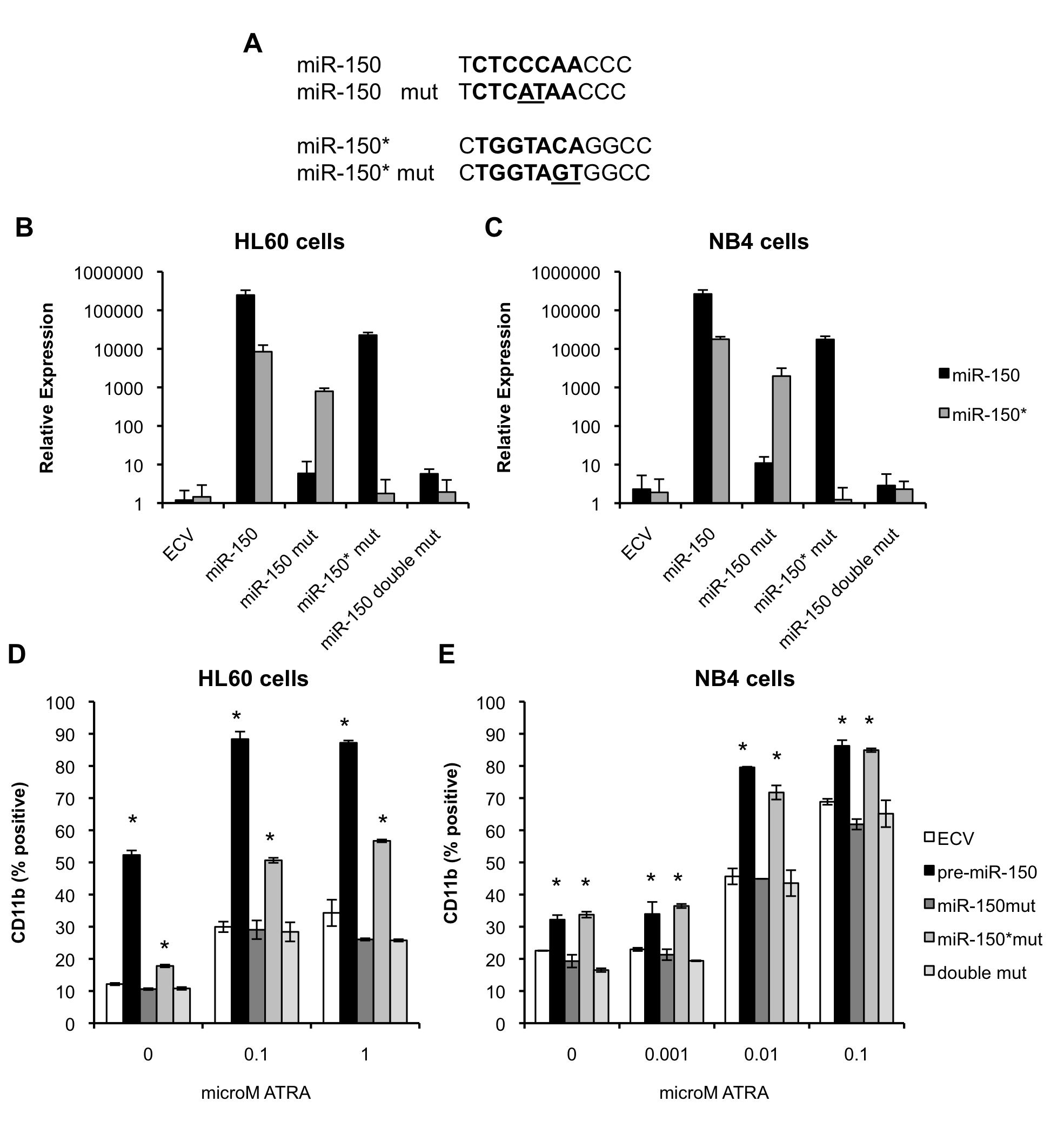

Supplement: Figure S5 — Myeloid differentiation in miR-150 expressing cells is mediated through the mature miR-150 strand. (A) Seed sequences of miR-150 mature or star strand were mutated in the pre-miR-150 expression vector as indicated. (B, D) HL60 cells and (C, E) NB4 cells were transduced with the various pre-miR-150 lentiviral vector constructs or empty control (ECV) lentivirus. Both mature miR-150 and miR-150* strands are expressed in pre-miR-150 expressing cells as measured by QPCR. Mutation of either miR-150 mature or star seed sequence resulted in absent or low expression of the mutated strand, but maintained expression of the opposite strand, although at a 10-fold decrease relative to the unmutated pre-miR-150 construct. (D) Transduced HL60 and (E) NB4 cells were assayed for CD11b expression by flow cytometry. Mutations in the mature miR-150 strand but not miR-150* abrogated miR-150 induced CD11b expression, indicating mature miR-150 targets mediate the differentiation phenotype. Two independent experiments, each with three technical replicates were performed; the means are shown and the error bars represent standard deviations (*P <0.05, Student’s t-test). (TIF) [file pone.0075815.s005.tif]

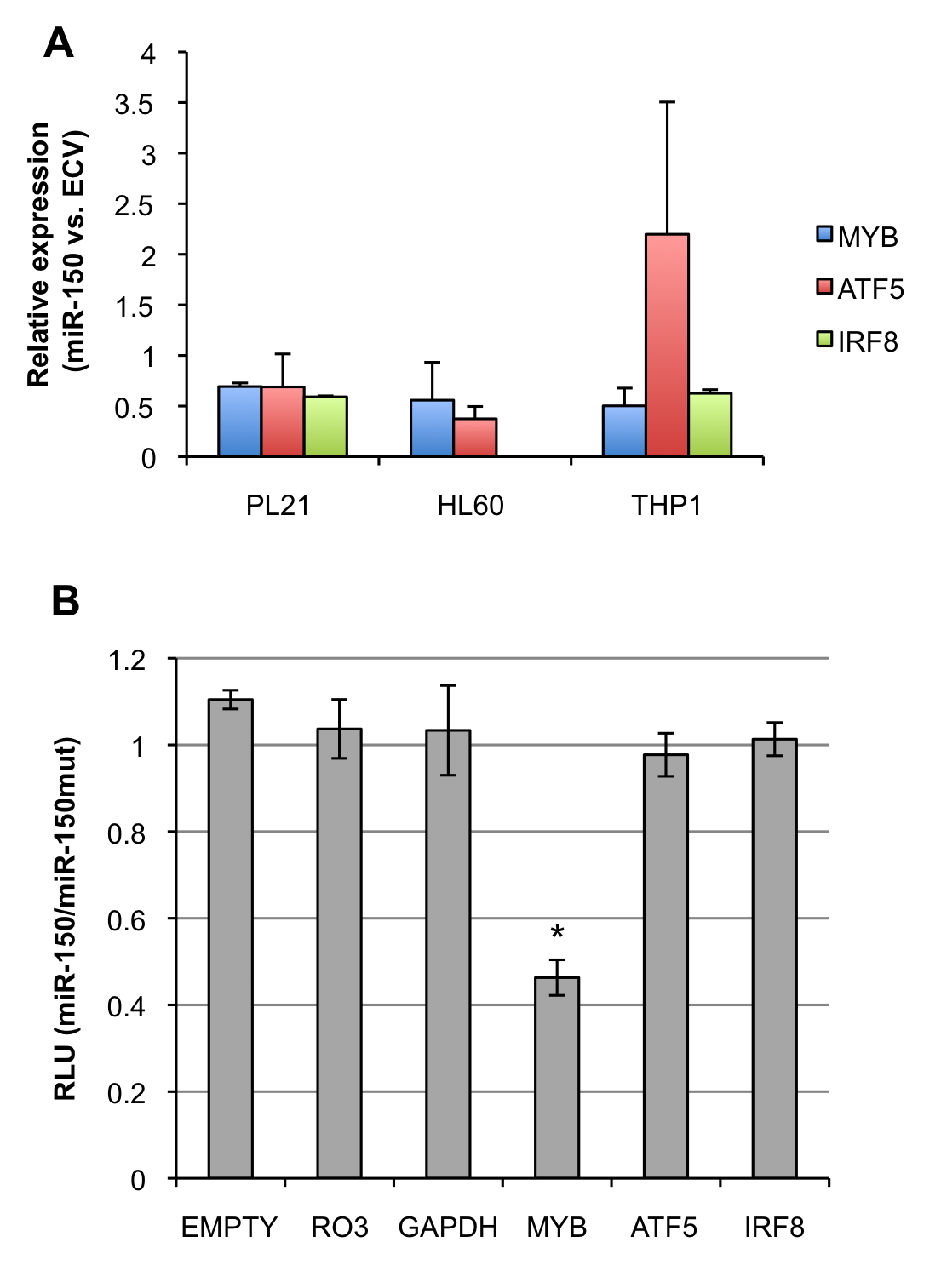

Supplement: Figure S6 — MiR-150 directly regulates the putative target MYB. (A) Expression of putative miR-150 targets, MYB, ATF5 and IRF8, was examined by QPCR in PL21, HL60 and THP-1 cells transduced with miR-150 versus empty control virus (ECV) 7-9 days after transduction. The relative fold-difference in expression for miR-150 vs. ECV cells is displayed. Three independent experiments, each with two technical replicates were performed; the means are shown and the error bars represent standard deviations No difference in expression between miR-150 vs. ECV cells is represented as 1. (B) K562 cells were co-transfected with 3'UTR LightSwitch Renilla luciferase reporters and pre-miR-150 or pre-miR-150 double mutant expression plasmids, and pGL3-Promoter Firefly luciferase as transfection control. GAPDH 3'UTR, random genomic sequence (RO3) 3'UTR, and empty vector served as negative controls. Relative luciferase units (RLU) represent Renilla/Firefly luminescence reads of the ratio of miR-150 vs. the double mutant miR-150 construct. Three independent experiments, each with three technical replicates were performed; the means are shown and the error bars represent standard deviations (*P≤0.005, Student’s t-test). (TIF) [file pone.0075815.s006.tif]
