# Supplementary material for: MicroRNA-150 Expression Induces Myeloid Differentiation of Human Acute Leukemia Cells and Normal Hematopoietic Progenitors
Source: PLoS One. 2013 Sep 24;8(9):e75815. doi: 10.1371/journal.pone.0075815 (PMC3782459; doi:10.1371/journal.pone.0075815)
Supplement: Methods S1 — (DOCX) [file pone.0075815.s007.docx]

**Supplemental Methods**

**Cell line and primary cell culture and transduction.** Our techniques are modified from our previous publication [1]. A multiplicity of infection (MOI) of 5 was used for cell lines and a MOI of 10 was used for primary cell lentiviral transduction in the presence of protamine sulfate (5 μg/mL, Sigma-Aldrich, St. Louis, MO, USA). Transduction efficiencies ranged from 50-85% for cell lines and from 20-40% for primary cells.

Primary patient samples were thawed in media containing Iscove’s Modified Dulbecco’s Medium (IMDM), 20% FBS, 2mM L-glutamine, 1% penicillin/streptomycin, 0.1 mM β-mercaptoethanol, 50 ng/ml each of rhSCF, rhIL3, rhIL6, rhGCSF, and rhGMCSF. Cells were plated at 500,000 cells per ml on CH-296 (retronectin) (Takara, Otsu, Shiga, Japan) coated-plates at 2 μg/cm^2^ at a density of 1.3-1.5 × 10^5^ cells per cm^2^ and cultured overnight at 37 degrees Celsius in a 5% carbon dioxide humidified incubator. A double transduction protocol was utilized for improved transduction efficiency. Cells were transduced for eight hours. After 8 hours the viral culture media was removed, suspended cells were spun down at 1,500 rpm for 10 minutes, and the viral culture media was replaced with fresh media overnight. The following day a second transduction was performed for 6 hours using fresh viral supernatant. Suspended cells were harvested and adherent cells were mobilized and all cells were spun down at 1,500 rpm for 10 minutes. Cells were resuspended in media at a concentration of 700,000 cells per ml on non-TC plates. Cells were sorted using GFP fluorescence 3 days after the first transduction. In order to examine the effects of miR-150 expression on *in vitro* myeloid differentiation, cells after sorting were resuspended at a concentration of 700,000 cells per ml. All patient samples but BC CML patient sample 2 were grown with the addition of 2 U/mL erythropoietin (PROCRIT®, Janssen Biotech, Inc., Horsham, PA, USA).

**Microarray studies.**

For all patient and cell line experiments 100 ng of high quality RNA was used. Total RNA was labeled and hybridized to Illumina Human HT-12 v.4 microarrays according to the manufacturer’s instructions by the FHCRC Genomics Core. Duplicates were performed for all experiments. Signal intensities were quantile normalized using the “lumi package” from Bioconductor (<http://www.bioconductor.org/>) and log_2_-transformed. GenePlus™ software (Enodar Biologic, Seattle, WA), which uses estimating equation techniques, was also used to determine differential expression between groups using ANOVA analysis to calculate p-values, z-scores, and the number of false discoveries [2]. Ingenuity Pathways Analysis (IPA, Ingenuity Systems®, www.ingenuity.com) was used to determine biologically enriched pathways and functions based on relationships in published literature. The p-value is calculated using a right-tailed Fisher's Exact Test and measures the statistical significance of a particular function or pathway in our data with respect to the reference set defined by IPA. For miRNA target analysis, targets are culled primarily from TargetScan (http://www.targetscan.org/), but also include other targets collated by IPA. The program allows for prediction of putative miRNA targets that are increased or decreased in our gene expression data sets. A new feature of IPA is the Upstream Regulator Analysis. Upstream regulators included transcription factors, cytokines, miRNAs, receptors, kinases, and chemicals and drugs. Upstream Regulator Analysis identifies putative upstream regulators that are activated or inhibited in our gene expression data sets. Activation or inhibition is assigned based on relationships derived from the literature and various databases and the direction of expression change in the array data provided. It also identifies candidates that are statistically significantly enriched when the direction cannot be ascertained. The p-value is calculated using Fisher’s Exact Test.

**RNA sequencing.** For pediatric AML patients, RNA was processed and sequenced by the BC Cancer Agency (Vancouver, BC, Canada). Using an adaptor that binds to the 3’ end of the RNA, PCR products are pooled and size-selected on a 96-channel robot to enrich the miRNA-containing fraction and to remove adapter contaminants. Each pool is then diluted to a target concentration for cluster generation and loaded into an Illumina Hiseq 2000 flow cell for sequencing.  Raw mature strand expression levels were presented as the number of reads aligned to a miRNA for each patient sample. This data is then normalized to reads per million and then logged to a base 2.

**Luciferase Assays.** 3’UTR LightSwitch luciferase reporters for GAPDH, MYB, ATF5, and Random Genomic Sequence (RO3) were purchased from SwitchGear Genomics (Menlo Park, CA, USA). IRF8 3’UTR containing one putative miR-150 binding site was cloned into the *Xba1* and *Xho1* sites of the 3’UTR pLightSwitch empty plasmid using PCR. HT1080 or K562 cells were co-transfected with pre-miR-150 or double mutant pre-miR-150 lentiviral constructs, and the pGL3-Promoter vector (Promega, Madison, WI, USA) as a transfection control and assayed using the Dual-Luciferase Reporter Assay (Promega) on a BioTek Synergy plate reader (BioTek, Winooski, VT, USA).

**References.**

References

1. Oehler VG, Guthrie KA, Cummings CL, Sabo K, Wood BL, et al. (2009) The preferentially expressed antigen in melanoma (PRAME) inhibits myeloid differentiation in normal hematopoietic and leukemic progenitor cells. Blood 114: 3299-3308. 10.1182/blood-2008-07-170282.

2. Zhao LP, Prentice R, Breeden L. (2001) Statistical modeling of large microarray data sets to identify stimulus-response profiles. Proc Natl Acad Sci U S A 98: 5631-5636. 10.1073/pnas.101013198.

3. Petriv OI, Kuchenbauer F, Delaney AD, Lecault V, White A, et al. (2010) Comprehensive microRNA expression profiling of the hematopoietic hierarchy. Proc Natl Acad Sci U S A 107: 15443-15448. 10.1073/pnas.1009320107.

4. Stegmaier K, Ross KN, Colavito SA, O'Malley S, Stockwell BR, et al. (2004) Gene expression-based high-throughput screening(GE-HTS) and application to leukemia differentiation. Nat Genet 36: 257-263. 10.1038/ng1305.
